# How standardized are “standard protocols”? Variations in protocol and performance evaluation for slow cortical potential neurofeedback: A systematic review

**DOI:** 10.3389/fnhum.2022.887504

**Published:** 2022-09-02

**Authors:** John Hasslinger, Micaela Meregalli, Sven Bölte

**Affiliations:** ^1^Center of Neurodevelopmental Disorders (KIND), Centre for Psychiatry Research, Department of Women's and Children's Health, Karolinska Institutet & Stockholm Healthcare Services, Region Stockholm, Stockholm, Sweden; ^2^Child and Adolescent Psychiatry, Stockholm Healthcare Services, Region Stockholm, Stockholm, Sweden; ^3^Curtin Autism Research Group, Curtin School of Allied Health, Curtin University, Perth, WA, Australia

**Keywords:** slow cortical potentials (SCP), neurofeedback, self-regulation, systematic review, brain computer interface (BCI)

## Abstract

Neurofeedback (NF) aims to alter neural activity by enhancing self-regulation skills. Over the past decade NF has received considerable attention as a potential intervention option for many somatic and mental conditions and ADHD in particular. However, placebo-controlled trials have demonstrated insufficient superiority of NF compared to treatment as usual and sham conditions. It has been argued that the reason for limited NF effects may be attributable to participants' challenges to self-regulate the targeted neural activity. Still, there is support of NF efficacy when only considering so-called “standard protocols,” such as Slow Cortical Potential NF training (SCP-NF). This PROSPERO registered systematic review following PRISMA criteria searched literature databases for studies applying SCP-NF protocols. Our review focus concerned the operationalization of self-regulatory success, and protocol-details that could influence the evaluation of self-regulation. Such details included; electrode placement, number of trials, length per trial, proportions of training modalities, handling of artifacts and skill-transfer into daily-life. We identified a total of 63 eligible reports published in the year 2000 or later. SCP-NF protocol-details varied considerably on most variables, except for electrode placement. However, due to the increased availability of commercial systems, there was a trend to more uniform protocol-details. Although, token-systems are popular in SCP-NF for ADHD, only half reported a performance-based component. Also, transfer exercises have become a staple part of SCP-NF. Furthermore, multiple operationalizations of regulatory success were identified, limiting comparability between studies, and perhaps usefulness of so-called transfer-exercises, which purpose is to facilitate the transfer of the self-regulatory skills into every-day life. While studies utilizing SCP as Brain-Computer-Interface mainly focused on the acquisition of successful self-regulation, clinically oriented studies often neglected this. Congruently, rates of successful regulators in clinical studies were mostly low (<50%). The relation between SCP self-regulation and behavior, and how symptoms in different disorders are affected, is complex and not fully understood. Future studies need to report self-regulation based on standardized measures, in order to facilitate both comparability and understanding of the effects on symptoms. When applied as treatment, future SCP-NF studies also need to put greater emphasis on the acquisition of self-regulation (before evaluating symptom outcomes).

**Systematic review registration:**

https://www.crd.york.ac.uk/prospero/display_record.php?ID=CRD42021260087, Identifier: CRD42021260087.

## Introduction

Neurofeedback (NF) is a form of brain-computer interface (BCI). Furthermore, it is an umbrella term used to describe different methods that provide feedback to an individual about neural activity, with the intent to enable the individual to consciously alter the activity. NF has been studied comprehensively over the past two decades as a treatment option for many conditions such as pain management (Roy et al., 2020), psychiatric disorders associated with criminal offending (Fielenbach et al., 2018), increased sports performance (Xiang et al., 2018), pediatric epilepsy (Nigro, 2019), migraine (Ambrosini et al., 2003), depression (Trambaiolli et al., 2021), and foremost attention-deficit hyperactivity disorder (ADHD) (Enriquez-Geppert et al., 2019).

Outcomes across conditions and studies have been inconclusive. Although pioneering studies have successfully shown that NF participants can achieve intentional regulation of specific brain activities (Birbaumer et al., 1981), Alkoby et al. (2018) found in their review that the portion of people who do not achieve regulation ranged between 16 and 57% of study participants. A recent systematic review addressed this issue of NF inefficacy, and synthesized the literature for predictors of successful self-regulation and beneficial post-treatment outcomes (Weber et al., 2020). The authors found seven predictors, with neurophysiological baseline parameters having strong predictive qualities (e.g. contingent negative variation). However, the interpretability was restricted due to the differences in study designs, NF protocols and outcome criteria.

It has been argued that most favorable outcomes in NF are attributable to unspecific effects, not closely connected to the method itself, such as believing in the treatment, interacting with the practitioner, the amount of positive feedback and the sense of control of one's brain signals (Thibault and Raz, 2017). Furthermore, studies that have implemented a placebo-controlled design (Arnold et al., 2013, 2021; Van Dongen-Boomsma et al., 2013; Vollebregt et al., 2014; Schönenberg et al., 2017) have consistently failed to show superiority of NF over sham conditions, with symptom improvements for both experimental and placebo conditions (e.g., feedback based on pre-recorded or randomly generated EEG). Others, in favor of NF have pointed out that methodological shortcomings and a lack of NF protocol standardization (Vollebregt et al., 2014; Van Doren et al., 2019) might account for such less favorable results. Specifically, researchers advocating for NF point out that sham-NF studies disregard important principals of operant conditioning, for instance by implementing high reward rates combined with frequent auto-thresholding, where successful self-regulation is “punished” by increases of success thresholds, while self-regulation failures are rewarded by lowering threshold, in order to maintain the set reward rate (Pigott et al., 2017). Furthermore, sham-NF usually neglects to demonstrate that the targeted self-regulation component has been learned by the participants (Pigott et al., 2021). Therefore, to assess self-regulation performance of the chosen NF-specific modality is of paramount importance when evaluating NF behavioral outcome. Unfortunately, the reporting of such data has often been neglected in previous research, why a recent consensus paper presented guidelines and a checklist covering both experimental design factors (e.g., blinding, employment of control conditions and measures) and the reporting of the specific NF modalities, including reinforcement schedules, strategies used, regulation success for both within- and between-sessions, as well as statistical analyses thereof (Ros et al., 2020).

One of the most researched NF protocols utilizes the regulation of slow cortical potentials (SCP), which are event-related-potentials that are either electrically negative or positive and last from several hundred msec. to several sec. SCP regulate cortical activity and prepare for physical and cognitive actions, in addition to regulating attention and memory (Birbaumer et al., 1990; Elbert, 1993; Birbaumer, 1999). A shift in increased negativity decreases the threshold for neural excitability and increases overall cortical activity, while a positive shift is associated with decreased excitability and inhibition (Birbaumer et al., 1990). The aim in Slow Cortical Potential-Neurofeedback (SCP-NF), is to learn to produce positive and negative shifts intentionally. The training usually consists of several trials that last for around 6–10 s. Each trial is preceded by a passive segment of around 2 s, which serves as baseline for the active phase during which the desired shift is generated, by either increasing or decreasing the cortical activity relative to the baseline value. The active phase is usually initiated by an acoustic signal together with the appearance of a prompting cue, that indicates in what direction the shift is to be steered. Often “up” indicates an increased activation (i.e., increased negativation), while “down” indicates decreased activation (i.e., increased positivation). The participants' performance is displayed in real-time on screen, e.g., via the altitude of an object that moves horizontally across the screen, based on an up/down modality. If the object is steered in the correct direction (as indicated by the cue), a reward animation is displayed, and the trial is deemed successful. As the electroencephalogram (EEG) signal is prone to artifacts, artifacts generated by muscle tension and eye movements, are corrected online via different algorithms (Strehl, 2009). In order to enable the transfer of self-regulation from the training setting into daily life, trials with delayed feedback are implemented. During such trials, the participant is only prompted with the start signal and the cue, but is not receiving any contingent on-screen feedback. However, the reward is displayed if the trial was successful. To further facilitate the transfer into daily-life, cards with pictures from the training screen are utilized to assist “dry runs” outside of the lab/clinic.

It is well-established that healthy and neurotypical individuals can learn to intentionally self-regulate positivation and negativation shifts (Elbert et al., 1980; Lutzenberger et al., 1980). However, studies have indicated that individuals with schizophrenia (Schneider et al., 1992b), and alcohol substance abuse (Schneider et al., 1993) have impaired self-regulatory control, although when staying sober for a long period, they can achieve successful regulation of their SCPs. Similarly, individuals with depression show successful regulation (Schneider et al., 1992a). SCP-NF has been utilized as a treatment for several conditions such as epilepsy (Kotchoubey et al., 2001), migraine (Siniatchkin et al., 2000a), tinnitus (Milner et al., 2016), and ADHD (Aggensteiner et al., 2019). It has been postulated that the self-regulation of SCPs may impact the sleep-spindle circuitry and thereby improve sleep (Arns and Kenemans, 2014; Arns et al., 2014), which may be beneficial for a multitude of conditions. Although the etiology of these conditions may vary, the implementation of SCP-NF is rather comparable, with only the ratio of negative to positive shifts differing dependent on the condition, why SCP-NF have sometimes been considered as a one-size-fits-all NF method (Mayer et al., 2013). Independent of the assumed mechanism-of-action, improvements in clinical symptoms seem to be related to successful self-regulation (Mayer et al., 2013), and their active implementation into everyday life (Strehl et al., 2005). Contrary to most NF protocols, which can be interpreted within a traditional “conditioning-and-repairing model,” SCP-NF is best understood within a “skill-acquisition model,” requiring effortful learning of the regulation-skill (For a detailed description of these models see (Gevensleben et al., 2014c).

Unfortunately, it is not uncommon that SCP-NF studies do not adequately report self-regulation performance. Furthermore, there is no uniform operationalization of self-regulation, limiting comparability of success rates between studies. Moreover, although SCP-NF is seen as a “standard protocol” (Arns et al., 2014), between studies there are many variations in the protocols' details (e.g., number of sessions, trials, trial length, utilization of transfer trials, etc.). Overall, little is known about how different protocol parameters may influence the ability to learn self-regulation, as well as effect outcome measure. Hence, “standard protocols” are not necessarily *standardized*-protocols. Such terminological unclarities are ethically challenging, as they may misguide stakeholders and give the false impression of being a gold standard, which is particularly problematic in face of the resource demands of NF in terms of time and costs. Also, commercially available NF, often lacks nuance between different protocols, and potentially overstates the benefits of NF. Hence, thoroughly assessing the intricacies of specific NF protocols is warranted.

Contrary to the review by Weber et al. (2020), we sought to also review publications that focus on a Brain-Computer-Interface (BCI) application, in particular those concerning the Thought Translation Device. This is a device that utilizes SCP regulation as a means of communication, via simple binary choices, e.g., the selection of letters, and is primarily intended for use in completely paralyzed locked-in patients, and those with amyotrophic lateral sclerosis, ALS (Birbaumer and Rodden, 2007). Since a high rate of correct differentiation is of pivotal importance for this type of application, we believe it may provide insights relevant for the clinical application of SCP-NF as well.

In summary, despite efforts to increase both the quality and quantity of reported regulation data in NF (i.e., CRED-nf), standards regarding the operationalization of self-regulation success do not exist. At the same time, meta-analyses indicate that when only considering “standard protocols,” such as SCP-NF, more outcomes are significant and effect sizes are greater (Cortese et al., 2016; Van Doren et al., 2019; Riesco-Matías et al., 2021). Therefore, illuminating potential differences in SCP protocols is important for future standardizations of this common (standard) protocol. Furthermore, successful self-regulation in NF constitutes the premise for positive outcomes. How the self-regulation skill has been evaluated and promoted is therefore key for understanding the development of self-regulation in SCP-NF. The purpose of this paper was therefore to systematically review differences concerning protocol-details and differences in how success in SCP self-regulation has been evaluated in SCP-NF studies so far, including rates of successful regulators. Finally, our aim was to synthesize the above and provide suggestions for SCP-NF that is implemented in clinical practice.

## Methods

### Registration and search strategy

We conducted a systematic review following the PRISMA statement criteria (Moher et al., 2016). The review protocol was registered on PROSPERO (registration number: CRD42021260087). In order to limit the risk of missing relevant publications, we implemented a broad search strategy based on “slow cortical potential^*^” as pivotal search term, with no limitations on language, year or type of publication. Searches were conducted in: Medline (Ovid), Web of Science (Clarivate), PsycInfo (Ovid), and ERIC (ProQuest), by experienced librarians at the University Library of Karolinska Institutet. See the Supplementary material for exact searches per database (Supplementary Table S1).

### Article selection and assessment

**Figure 1:**
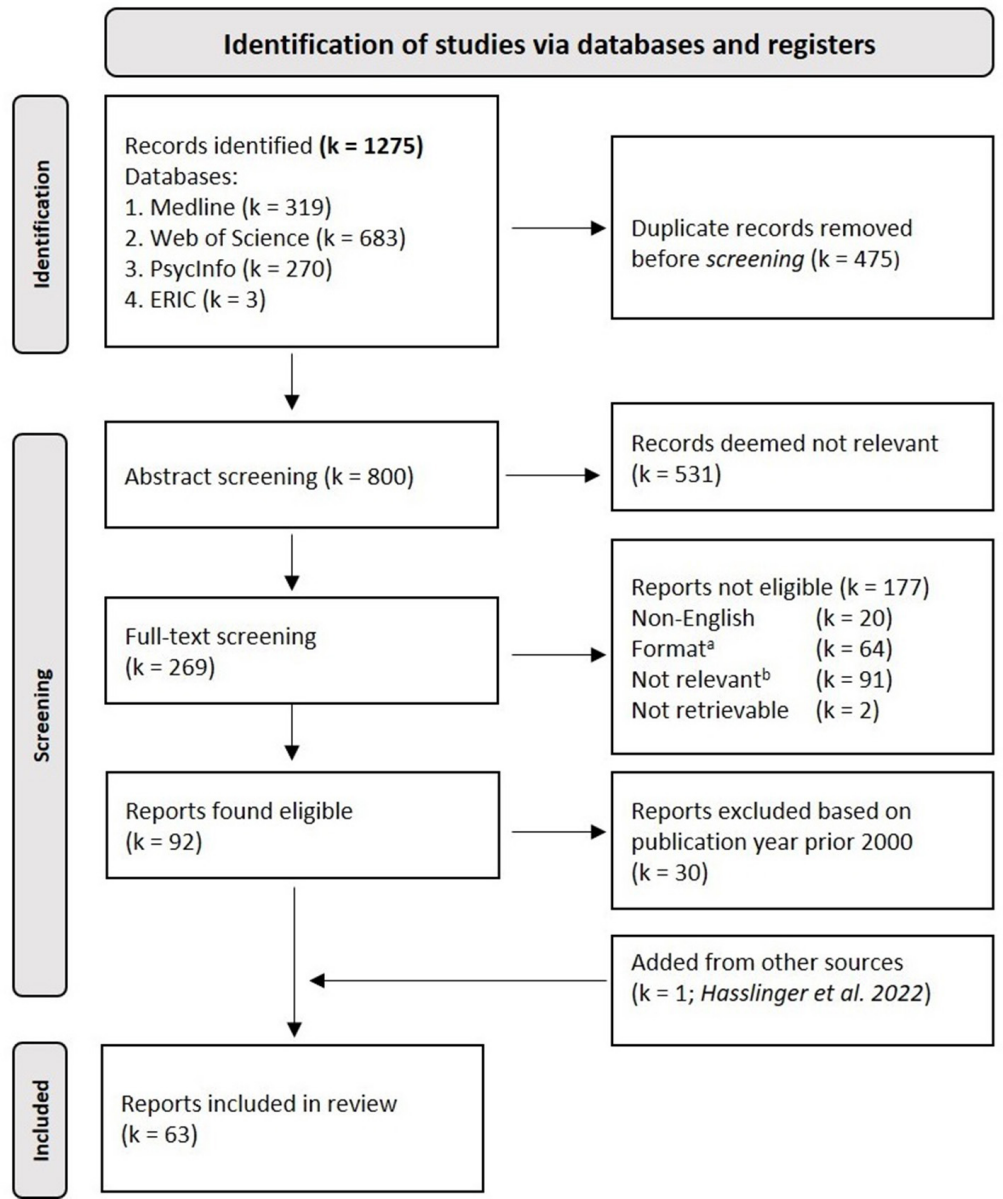
PRISMA flow-diagram. ^*a*^e.g., Conference abstracts, Books and Book-chapters; ^*b*^e.g., Reviews, Editorials and Non-relevant experiments.

The literature search rendered 1,275 articles. After eliminating 475 duplicates, the titles and/or abstracts of 800 articles were screened independently by two authors (JH, MM). Articles that both authors deemed not relevant (e.g., animal studies, theoretical contributions, reviews, etc.) were excluded (k = 531). Full texts of articles that at least one of the authors deemed eligible, were included in the full text assessment (*k* = 269), which were reviewed independently by two authors (JH, MM). Any discordance regarding inclusion was discussed between researchers until consensus was reached. Only original articles that were available in English were finally included. A report from our own RCT (Hasslinger et al., 2022), that had not yet been published at the time of the literature search was also added. In total *k* = 93 articles met our eligibility criteria and were included. However, in order to maintain the study results up-to-date and relevant for future endeavors, we decide to only include articles published from the year 2000 onwards for the final review, leaving *k* = 63 articles for the current literature synthesis. The process is illustrated in the flowchart (see Figure 1). For transparency, eligible articles published prior the year 2000 are presented in the Supplementary Table S2.

For increased clarity, articles that utilized SCP self-regulation in a BCI context (*k* = 14), and articles that reported studies that employed SCP-NF in a therapeutic context (*k* = 49) are presented separately. Since the impetus of this review is to provide an overview to aid the evaluation of successful SCP self-regulation and future protocol designs for clinical application, the focus will be on these areas.

Besides of assessing variables for the number of participants, their age and medical status (i.e., disorder/diagnosis), we wanted to assess details concerning the SCP-NF training protocols. We therefore extracted electrode placement and what equipment was used as well as the number of sessions, number of runs/blocks, number of trials, the trial length/duration (baseline and active phase), the use of thresholds, the ratio of positivation (deactivation) to negativation (activation) trials, the use of transfer trials, and the use of transfer-promoting exercises (e.g., transfer card). Furthermore, as our main area of interest lays with self-regulation, we extracted information concerning the definition of successful regulation, how regulators were categorized, and the outcomes for successful regulators.

In order to appraise the quality of the 49 articles that studied SCP-NF within a clinical setting (i.e., not BCI studies), two authors (JH, MM) independently evaluated each article according to the CRED-nf checklist independently. Discordance on items were discussed until consensus between the authors was reached. Details concerning scoring criteria are presented in the Supplementary Table S2.

## Results

Nine studies had multiple articles published. In these cases, only one published report is referred to in order to improve readability. Overall, 34 studies in a clinical setting were reported in 49 articles, in addition 14 articles reported on 13 BCI-focused studies. Table 1 provides an overview over the protocol-details for these studies, and lists all articles linked to them.

**Table 1:** Summary of protocol-details of included studies.

**Study/articles (cited article is bolded)**	**Sample: [1] Diagnose; [2] Age; [3] Number of participants**	**Equipment & set-up [1] Active; [2] Eye-correction; [3] Reference; Ground**	**Training Volume: [1] Number of sessions; [2] Frequency; [3] Trials (blocks x trials) per session**	**Trial details: [1] Ratio; [2] Length; [3] Transfer trials; [4] Thresholds**	**Reward system**	**Transfer exercises**
Marx et al. (2015), Strehl et al. (2017), Aggensteiner et al. (2019)	[1] ADHD [2] *M* (*sd*) = 8.6 (0.92) [3] 72 (60 at FU)	EQ: Neuroconn [1] Cz [2] vEOG + hEOG, [3] Mastoids	[1] 25 (12 sessions – 4–6 weeks break – 13 sessions) [2] 2–3 sessions per week [3] 160 (4 × 40, FB; FB; TR; FB)	[1] **S1–12**: 50:50; **S13-25**: 20:80 [2] 2 s bl; 8 s active [3] TR: 1 block 100% [4] n/s	Tokens earned for participation and good cooperation. Tokens were exchanged for small gifts/vouchers.	[a] Transfer cards and DVDs were introduced during break. [b] For the last 10 sessions, homework was done in the lab after each session, using the cards, supervised by therapist.
Hasslinger et al. (2020, 2022)	[1] ADHD [2] Range = 9–17^*^ [3] 50	EQ: Neuroconn [1] Cz [2] vEOG + hEOG [3] Mastoids	[1] 25 (+2 at FU) [2] 5 per week [3] 144 (4x36)	[1] 50:50 [2] 2 s bl; 8 s active [3] TR: S1–5: 20%. S6–10: 40%. S11–25: 50%. [4] ± 40 μV	Tokens earned for conduct, exchanged to a voucher (~ €20). Also, separate voucher for participating in study (~ €50).	Transfer cards for daily use were introduced from session 10. Parents instructed to remind participant of task.
Minder et al. (2018), Zuberer et al. (2018)	[1] ADHD [2] Range = 8–15 [3] 54 (25 in school; 29 in clinic); 48 in Zuberer et al. (2018)	EQ: Neuroconn [1] Cz [2] n/s [3] Mastoids	[1] 15 [30 units] [2] Within 3 months [3] 160 (4x40)	[1] 50:50 [2] 2s bl, 8 s active (+ 2 reinforcement) [3] TR: **S1–2**: 20/20/20/20. **S3–5**: 20/20/20/40. **S6–8**: 20/20/40/40. **S9–13**: 20/40/40/50. **S14–15**: 50/50/50/50 [4] ±40 μV	Not specified/ not reported	Transfer strategies and use of transfer cards were implemented via parents or teachers *(Timepoint of introduction n/s.)*
Albrecht et al. (2017)	[1] ADHD [2] Range = 7–17 [3] 24 (13 at FU)	EQ: Neuroconn [1] Cz [2] vEOG + hEOG [3] Mastoid; n/s	[1] 20 [2] 5 sessions per week (2 weeks), 2 sessions per week (5 weeks) [3] S1-6: 96 (3 × 32). S7–15: 120 (3 × 40). S16–20: 150 (3 × 50)	[1] **S1–15**: 50:50; **S16–20**:40:60 [2] 2 s bl; 5.5s active [3] TR: **S1–6**: 0%. **S7–11**: 20%. S12–15: 50%. S16–20: 40% [4] 30% of 80μV = 24 μV	Tokens earned for successful sessions. (*Definition of a successful session not provided*.)	Transfer cards for daily use, in situations demanding relaxation or attentiveness, were introduced from session 10. A training log was kept.
Mayer et al. (2016)	[1] ADHD [2] Range = 18–60 [3] 24	EQ: Neuroconn [1] Cz [2] vEOG + hEOG [3] Mastoids	[1] 30 (15 sessions - 3 weeks break – 15 session) [2] Max 5 per week^a^ [3] 160 (4x40)	[1] 50:50 [2] 2 s bl; 8 s active [3] TR: 1 block 100% [4] n/s	No rewards.	Transfer cards and DVD (showing transfer session) introduced during break. Participants were instructed to document the use.
Okumura et al. (2019)	[1] ADHD [2] Range = 7–16 [3] 22	EQ: Neuroconn [1] Cz [2] n/s [3] Right earlobe; Forehead	[1] 10 (20 units) [2] ~ 1 per week [3] 60/unit	[1] 50:50 [2] 2 s bl; 8 s active [3] TR: 20% [4] Yes, level n/s	Not specified/ not reported	n/s
Christiansen et al. (2014)	[1] ADHD [2] *M* (*sd*) = 8.42 (1.34)^*^ [3] 14 (preliminary data, 58 whole sample)	EQ: Neuroconn [1] Cz [2] vEOG + hEOG [3] Mastoid; n/s	[1] 30 (12 sessions−1 week break−12 sessions−1 week break−6 sessions) [2] 3 sessions per week [3] 120 (3x40)	[1] S1–24: 50:50; S25–30:25:75 [2] 2 s bl; 6 s active [3] TR: 1 block 100% [4] Introduced each session when correct responses = ≥70%, 5% initial threshold and increased by 5% at a time if the correct responses = ≥70%,	Per session, participants could earn up to 5 tokens, if attentive for the whole session (15 tokens = small rewards).	Regulation strategies were identified with trainer, used daily in attention demanding situations. Documented daily by participant and controlled at each session *(Timepoint of introduction n/s)*
Takahashi et al. (2014)	[1] ADHD [2] Range = 8–16 [3] 10	EQ: Neuroconn [1] Cz [2] n/s [3] Ear lobe; Forehead	[1] 20 (16 used in analysis) [2] 2 session per week [3] 60 (1x60)	[1] 50:50 [2] 2s bl; 8s active [3] TR: 20% [4] “Target level” ±30μV to ±50μV	Not specified/ not Reported	n/s
Mayer et al. (2012)	[1] ADHD + healthy controls [2] ADHD: *M (sd)* = 28.4 (3.83); Controls: *M (sd)* = 28.4 (3.83) [3] 10	EQ: Neuroconn [1] Cz [2] vEOG + hEOG [3] Mastoids	[1] 15 [2] 1–3 per week [3] 160 (4 × 40)	[1] 50:50 [2] 2s bl; 8s active [3] TR: 25% [4] n/s	Not specified/ not reported	Participants were instructed to apply regulation skills in everyday situations. *(Timepoint of introduction n/s)*
Krepel et al. (2020)	[1] ADHD [2] *M (sd)* = 24 (14.6), for whole 2019 sample [3] 9	EQ: Neuroconn [1] n/s [2] n/s [3] n/s	[1) n/s [2] 2–3 sessions per week [3] n/s	[1] n/s [2] n/s [3] n/s [4] n/s	Not specified/ not reported	n/s
Baumeister et al. (2018, 2019)	[1] ADHD [2] Range = 9–14^*^ [3] 8	EQ: Neuroconn [1] Cz [2] n/s [3] Mastoid; n/s	[1] 20 (10 sessions−14 days break−10 sessions) [2] 2–3 sessions per week [3] 160 (4 × 40: FB; FB; TR; FB)	[1] S1–10: 50:50; S11–20: 20:80 [2] 2s bl; 8s active [3] TR: 1 block 50 % (from 30–70%) [4] n/s	Points earned for each successful training block and for compliance. *Definition of a successful training block not provided*.	[a] “Short transfer exercises” in everyday life were introduced during break. [b] For the last 10 sessions, homework was done directly after training, applying SCP regulation skills, supervised by trainer.
Konicar et al. (2015, 2021b)	[1] Psychopathy [2] *M (sd)* = 43.14 (11.52) [3] 14	EQ: Neuroconn [1] FCz [2] vEOG+ hEOG [3] Mastoids	[1] 25 (12 sessions−13 days break−13 sessions) [2] Daily [3] 120 (3 × 40: FB; TR; FB)	[1] S1–12: 50:50; S13–25: 20:80 [2] 2s bl; 8s active [3] TR: 1 block 100% [4] n/s	Participants were compensated financially for their participation (€100).	Transfer cards were introduced during break.
Konicar et al. (2021a)	[1] ASD [2] *M (sd)* = 14.05, (1.76) [3] 21	EQ: Neuroconn [1] FCz [2] vEOG + hEOG [3] Mastoids	[1] 12 sessions−7 days breaks−12 sessions [2] Within 3 months [3] 120 (3 × 40)	[1] S1–12: 50:50; S13-24; 20:80 [2] 2 s bl; 8 s active [3] TR: 1 block 100% [4] n/s	Not specified/ not reported	Transfer cards for home use were introduced during break. Participants were instructed to document this in a “structured home training diary.”
Fumuro et al. (2013)	[1] Parkinson + healthy [2] Parkinson: Range = 36–71 Healthy: Range = 60–69 [3] 10 + 11	EQ: Neuroconn [1] Cz [2] vEOG + hEOG [3] Mastoid; n/s	[1] 2–4 [2] 1–6 days between sessions [3] 2–5 × 52	[1] 50:50 [2] 2s bl; 8s active [3] TR: 50% [4] Threshold ±40μV	Not specified/ not reported	n/s
Gevensleben et al. (2009a; 2009b^b^, 2010), Wangler et al. (2011), Heinrich et al. (2020)	[1] ADHD [2] Range = 8–12^*^ [3] 59 (38 at FU)	EQ: SAM (Self-regulation and Attention Management) [1] Cz [2] vEOG^c^ [3] Mastoid; n/s.	[1] 9 (18 units) [2] 2–3 sessions per week [3] ~120	[1] 50:50 [2] 2 s bl; 6 s active [3] TR: ~ 40–60% [4] n/s	Not specified/ not reported	From session 8, participants were instructed to practice regulation strategies in everyday situations for 10 min every day. The practice was documented and discussed during next session. Parents support was encouraged.
Gevensleben et al. (2014b)	*Study 1:* [1] ADHD [2] Range = 10–13 [3] 10 *Study 2:* [1] Tourette syndrome [2] Range = 9–16^*^ [3] 16	*Study 1:* EQ: SAM (Self-regulation and Attention Management) [1] Cz [2] vEOG [3] Earlobe; n/s *Study 2:* EQ: SAM (Self-regulation and Attention Management) [1] Cz [2] vEOG [3] Earlobe; n/s	*Study 1:* [1] 13 (26 units) [2] 1–3 per week [3] 4 x 36–48 *Study 2:* [1] 6–8 (18–24 units) [2] 3–4 days per week (for 2 weeks) [3] 7–8 x 30–40	*Study 1:* [1] n/s [2] 2 s bl; 6 s active [3] Yes, ratio n/s [4] n/s *Study 2:* [1] n/s [2] 2 s bl; 6 s active [3] TR: 30% [4] n/s	*Study 1:* Tokens earned for successful regulation. *Definition of successful regulation is not provided. Study 2*: Not specified/ not reported	*Study 1:* From session 6, “dry runs” and identifying situations for regulation strategies, were practiced at home. Parents attended NF-sessions to facilitate support for transfer. *Study 2:* From week 2, “dry runs” and identifying situations for regulation strategies, were practiced at home.
Studer et al. (2014)	[1] No clinical diagnosis [2] Range = 19–31^*^ [3] 19	EQ: SAM (Self-regulation and Attention Management) [1] Cz [2] vEOG [3] Mastoid; n/s	[1] 10 [20 units] [2] ~ 2 per week [3] 40–60 per block	[1] 50:50 [2] 2 s bl; 6 s active [3] TR: ~ 40% [4] n/s	Not specified/ not reported	From 5th double-session participants applied their strategies to attention-demanding tasks during last 10 min of session. Participants were instructed to do daily practice of regulation strategies in specific everyday situations.
Gevensleben et al. (2014a)	[1] Healthy adults [2] Range = 18–29^*^ [3] 9	EQ: SAM (Self-regulation and Attention Management) [1] Cz [2] vEOG [3] Mastoid; n/s	[1] 8 (16 units) [2] 2–3 per week [3] ~120	[1] 50:50 [2] 2 s bl; 6 s active [3] TR: >33% [4] n/s	Participants were compensated financially for their participation (€85).	n/s
Heinrich et al. (2004)	[1] ADHD [2] Range = 7–13 [3] 13	EQ: “GoeFI” (Goettinger Feedback) [1] Cz [2] vEOG [3] Mastoid; n/s	[1] 25 [2] Within 3 weeks [3] 120	[1] 50:50 [2] 2 s bl; 6 s active [3] TR: 33–50% [4] n/s	Not specified/ not reported	Participants were instructed to practice strategies at home in certain situations, starting from week 2. Practice was to be documented in a protocol.
Drechsler et al. (2007), Doehnert et al. (2008)	[1] ADHD [2] Range = 9–12 [3] 14 (17)	EQ: “GoeFI” (Goettinger Feedback) [1] Cz [2] vEOG + hEOG [3] Mastoid; n/s	[1] 15 (10 sessions, 5 weeks break, 5 sessions) [30 units] [2] First 10 in 2 weeks, then 5 sessions [3] 180 (40FB; 30TR; 40TR; break; 40FB; 30TR)	[1] 50:50 [2] 2 s bl; 6 s active [3] TR: 3 blocks 100% (of which one with transfer with cards) [4] n/s	Parents were instructed to reward training efforts (transfer exercises) with tokens. Tokens could be exchanged for small gifts	During the break, transfer cards were introduced, and participants had to practice their regulation strategies in everyday situations, that had been identified together with trainer. A practice dairy was used, and parent support and supervision were encouraged.
Strehl et al. (2006a), Leins et al. (2007)	[1] ADHD [2] Range = 8–13 [3] 23 (19)	EQ: EEG 8 (Contact Precision Instruments, Cambridge, MA) [1] Cz [2] “Corrected online for eye movements” [3] Mastoid; n/s	[1] 30 (10 sessions – 4–6 weeks break – 10 sessions −4–6 weeks break – 10 sessions) + 3 (FU) [2] 5 per week [3] 3–5 × 38–39	[1] S1–15: 50:50; S16-30–25:75. [2] 2 s bl; 6 s active ^d^ [3] TR: 23% [4] n/s	Tokens earned for each successful trial. Tokens were exchanged for small gifts (value ~€1.5).	[a] Transfer cards were introduced during breaks, and during the third training phase. Activation-strategies were practiced in situations where attention was required. Practice was to be documented. [b] After each session in the last training phase, participants trained activation
						strategies while doing homework in the lab, supervised by the trainer.
Kotchoubey et al. (2001), Strehl et al. (2005, 2011, 2014)	[1] Epilepsy [2] Range = 14–55^e^ [3] 41 (34 at 1y-FU; 16 at 8y-FU)	EQ: Neurofax amplifier + Neuroconn at 8y-FU [1] Cz [2[vEOG [3] Mastoid; n/s	[1] 35 (20 sessions−8 weeks break−15 sessions) + 3 at 8y-FU [2] 20 in 3 weeks + 15 in 2 weeks + 3 in 1 week [3] Blocks: S1–35: n/s; S36–38: 5 (FB; TR; FB; TR; FB) Trials: S1–35: 145; S36–38: 140	[1] S1–20: 50:50; S21–35: 67:33; S36–38: 60:40 [2] 2 s bl; 8s active [3] TR: S36–38: 2 blocks with 100% (2x 20 trials) [4] n/s	Not specified/ not reported	[a] Learned strategies were practiced at home during break. [b] Parallel to the SCP-NF, 15 sessions of behavioral therapy, aimed to facilitate self -regulation and its implementation into everyday life, were implemented.
Morales-Quezada et al. (2019)	[1] Epilepsy [2] *M (sd)* = 14.8 (2.3) [3] 16	EQ: ProComp InfinitiEncoder + EEG-Z3™ Sensor [1] Cz [2] vEOG [3] Earlobe; n/s	[1] 25 [2] 5 per week [3] 75	[1] 50:50 (trials 1–15); 67:33 (trials 16–75) [2] 2 s bl; 6 s active [3] n/s [4] n/s	Not specified/ not reported	Not specified/ not reported
Uhlmann and Fröscher (2001)	[1] Epilepsy [2] *M (sd) =* 38.5 (10.1)^*^ [3] 10	EQ: n/s [1] n/s [2] n/s [3] n/s	[1] 35 [2] Within 3 months [3] 145	[1] n/s [2] 8s [3] n/s [4] +10 μV (pos trials); −15 μV (neg trials).	Not specified/ not reported	Not specified/ not reported
Milner et al. (2016)	[1] Tinnitus [2] 50 [3] 1	EQ: Biograph Infinity 5.0. software [1] Cz [2] vEOG^f^ [3] Mastoid; C7^g^	[1] 30 (10 sessions−1 month break−10 sessions- 1 month break−10 sessions [2] Within 3.5 months [3] 160 (3 × 40FB + 1 ×40TR)	[1] S1-10: 1:1; S11-30: 2:1 (fixed order) [2] n/s [3] TR: 1 block 100 % [4] Individualized threshold targeting a 30% success rate. Separate threshold for activation/ deactivation, adjusted every session.	No rewards.	Transfer cards were introduced and used during breaks after session 10 and 20. Patients were instructed to imagine a training session, sitting in front of a PC, using the transfer card.
Siniatchkin et al. (2000a)	[1] Migraine [2] *M* = 10.5 (1.5) [3] 10	EQ: Nihon Kohden amplifier [1] Cz [2] vEOG [3] Mastoid; n/s	[1] 10 [2] 10 sessions in 8 weeks [3] 2 × 30 (30 × FB-; 30 × FB+) + 2 × 15 (15 × TR-; 15 × TR+)	[1] 50:50 (fixed order) [2] 2s bl; 3s active [3] TR: 33% (100% for 2 blocks of 15 trials) [4] Based on previous session.	Tokens earned when reaching criterion for successful regulation during repeated trials. For every 10 points, the participant was rewarded with a sweet.	Not specified/ not reported
Hinterberger et al. (2003)^¤^	[1] EP + healthy [2] Range = 19–52 [3] 10	EQ: n/s [1] Cz [2] vEOG [3] Mastoid; n/s	[1] EP: 35 (20 sessions – 8 weeks break – 15 sessions). Healthy: 10 [2] EP: 20 in 3 weeks + 15 in 2 weeks. Healthy: 10 in 2–weeks [3] 145; (90 in fMRI)	[1] n/s; in fMRI 1:1:1 ^#^ [2] 8s (0.5s bl) [3] TR: Yes, n/s further [4] n/s	Not specified/ not reported	Participants with epilepsy were instructed to practice learned strategies at home during break after session 20.
Kleinnijenhuis et al. (2008)	[1] Healthy [2] n/s (adults) [3] 9	EQ: Brainquiry PET-EEG [1] Cz [2] vEOG [3] Mastoid; AF3	[1] 20 [2] Over 8 weeks [3] 160 (4 × 40)	[1] 60:40 [2] 0.5s bl; 7s. active [3] TR: None. [4] Individualized thresholds, targeting an initial 33% success rate.	The participant was informed about the regulation success and socially rewarded by the trainer.	Not specified/ not reported
Kotchoubey et al. (2000)	[1] Healthy [2] Younger group: Range = 22–30; Older group: Range = 50–64 [3] 27 (15+12)	EQ: Neurofax (Nihon Kohden) amplifier [1] Cz [2] vEOG [3] Mastoid; n/s	[1] 4 [2] Daily [3] 144	[1] n/s [2] 2 s bl; 8 s active [3] 62/144 [4] n/s	Participants earned 15DM per hour + a bonus of 20DM for every session were a differentiation of minimum 5 μV was performed.	Not specified/ not reported
Kotchoubey et al. (2002)	*Study 1:* [1] EP [2] Range = 21–45 [3] 22 *Study 2:* [1] ALS [2] 33 [3] 1	*Study 1:* EQ: n/s [1] Cz [2] Yes, n/s [3] Mastoid; n/s *Study 2:* EQ: n/s [1] n/s [2] n/s [3] n/s	*Study 1:* [1] 35 (20 sessions – 8 weeks break ‘– 15 sessions) [2] First 20 sessions: Daily. Last 15 sessions: n/s [3] 140 *Study 2:* [1] n/s [2] 3 days per week [3] 1,000 (10 × 100)	*Study 1:* [1] n/s [2] 8s (bl and active n/s) [3] TR: 50–70 trials (increased based on performance) [4] n/s *Study 2:* [1] n/s [2] 2.5 s (bl and active n/s) [3] TR: None [4] n/s	*Study 1:* Not specified/ not reported *Study 2:* Not specified/not reported	Study 1: Participants were instructed to practice learned strategies at home during break. *Study 2:* n/s
Pulvermüller et al. (2000)	[1] Healthy [2] Range = 19–66 [3] 12	EQ: Nihon Kohden amplifier [1] C5 [2] vEOG + hEOG [3] Mastoid; n/s	[1] 12–20 [2] n/s [3] 100 (40FB; 30TR; 30FB)	[1] n/s [2] 1 s bl; 8 s active^h^ [3] TR: 30% [4] n/s	Participants were paid 15 DM per session.	n/s
Siniatchkin et al. (2000b)	[1] Healthy [2] *M (sd)* = 11.6 (1.2) [3] 9	EQ: n/s [1] Cz [2] vEOG [3] Mastoid; n/s	[1] 5 [2] Within 3 weeks [3] 60 (30 × FB-; 30xFB+)	[1] 50:50 (fixed order) [2] 2s. bl; 3s active [3] TR: None [4] Based on previous session	Tokens earned when reaching criterion for successful regulation during repeated trials. For every 10 points, the participant was rewarded with a sweet.	Children kept strategy-dairy, and were encouraged to practice strategies in everyday situations.
Spronk et al. (2010)	[1] Healthy [2] Range = 18–40^*^ [3] 9	EQ: Brainquiry PET-EEG [1] Cz [2] vEOG [3] Mastoid; AF3	[1] 20 [2] Over 8 weeks [3] 160 (4 × 40)	[1] 60:40 [2] 0.5s bl; 7s active [3] TR: None [4] Individualized thresholds, targeting an initial 33% success rate.	Not specified/ not reported	n/s
Strehl et al. (2006b) ^¤^	[1] Epilepsy [2] Range = 28–41 [3] 5	EQ: Neurofax (Nihon Kohden) amplifier [1] Cz [2] vEOG [3] Mastoid; n/s	[1] 1 [2] Not applicable [3] 200 (2x100)	[1] n/s ^#^ [2] 8s [3] TR: 100% [4] n/s	Not specified/ not reported	n/s
Birbaumer et al. (2000)	[1] ALS [2] n/s [3] 5 (of which 2 discontinued)	EQ: TTD, language support program [1] Cz [2] vEOG [3] n/s; Mastoid	[1] Depended on performance [2] Several times per week [3] 6–12 × 70–100	[1] n/s [2] 2 s bl, 2–4 s active [3] n/s [4] 5 μV, gradually increased to 8 μV	Not specified/ not reported	n/s
Kaiser et al. (2001)	[1] ALS [2] 43 and 31 [3] 2	EQ: TTD [1] n/s [2] n/s [3] n/s	[1] Depended on performance [2] Depended on performance [3] 70–100 per block, blocks n/s	[1] n/s [2] 2 s bl, 3 s active [3] TR: Feedback gradually omitted [4] n/s	Not specified/ not reported	n/s
Kübler et al. (2001)	[1] ALS [2] 45 and 31 [3] 2	EQ: TTD, Language support program [1] Cz, [2] vEOG [3] Mastoid; n/s	[1] 2–3 × 8 weeks [2] 2–3 training days per week [3] 10–20 x 70	[1] n/s [2] 2 s bl, 2.5–4 s (depending on patient's performance) active [3] n/s [4] Trial invalid if the amplitude average = ± 0.5 μV	Not specified/ not reported	n/s
Kaiser et al. (2002)	[1] ALS [2] 43 [3] 1	EQ: TTD, Language support program [1] Cz [2] vEOG [3] Mastoid; Forehead	[1] Data reported from 53 training days [2] Initial 3 months: On average: 6 days/month. Subsequent 2 years: 1.8 days/ month [3] 7–12 × 70	[1] n/s [2] 2 s bl, 3 s active [3] n/s [4] Trial invalid if the amplitude average = ± 0.5 μV	Not specified/ not reported	n/s
Neumann and Birbaumer (2003)	[1] ALS [2] Range = 31–66 [3] 5	EQ: TTD [1] Cz [2] vEOG [3] n/s; Mastoid	[1] Depended on performance [2] 2–4 times per week [3] 50–70 per block, blocks n/s	[1] n/s [2] 1–4s bl, 3–5 s active (adapted to patients) [3] n/s [4] Trial invalid if the amplitude average = ± 0.5 μV	Not specified/ not reported	n/s
Neumann et al. (2003)	[1] ALS [2] 47 (born 1955) [3] 1	EQ: TTD, “Language program” [1] Cz [2] vEOG [3] FPz; n/s	[1] n/s [2] 2–3 times × week. [3] 10–20 x 70–100	[1] n/s [2] 2 s bl, 2.5 active [3] TR: After a few weeks: feedback only provided during 500 ms of active phase [4] 7.7 μV	Not specified/ not reported	n/s
Kübler et al. (2004)	[1] ALS + healthy (no diagnose) [2] ALS: Range = 35–63 Healthy: Range = 18–39 [3] 10 + 10	EQ: TTD, Language support program [1] Cz [2] vEOG [3] Mastoid; n/s	[1] ALS: Depended on performance. Healthy: 6 [2] ALS: Once per week. Healthy: Two times per week [3] ALS: 2–12 x 50 Healthy: 10x50	[1] 50:50 [2] 2 s bl, 4 s active [3] n/s [4] Trial invalid if the amplitude average = ± 0.5 μV	Healthy controls were paid 8 euro/h.	n/s
Neumann et al. (2004)	[1] ALS [2] n/s [3] 2	EQ: TTD, Language support program [1] Cz [2] vEOG [3] Mastoid; n/s	[1] 23 vs. 31 training days [2] Individualized frequency, at least 2/week [3] n/s x 70	[1] n/s [2] 2 s bl, 3 s active [3] n/s [4] Trial invalid if the amplitude average = ± 0.5 μV	Not specified/ not reported	n/s
Hinterberger et al. (2005a)	[1] ALS [2] 58 [3] 1	EQ: n/s [1] Cz [2] n/s [3] Mastoid; n/s	[1] 3 [2] n/s [3] n/s	[1] n/s [2] n/s [3] n/s [4] n/s	Not specified/ not reported	n/s
Karim et al. (2006)	[1] ALS [2] n/s [3] 1	EQ: TTD, Descartes [1] Cz [2] Yes, n/s [3] n/s; n/s	[1] n/s [2] n/s [3] 2-3 x 100	[1] n/s [2] 2 s bl, 2 s active [3] none^i^ [4] 7 μV for FB+, no threshold for FB-.	Not specified/ not reported	n/s
Hinterberger et al. (2004b) ^¤^	[1] Healthy (no diagnose) [2] n/s [3] 3	EQ: TTD [1] Cz [2] vEOG [3] Mastoid; n/s	[1] One fMRI session (participants had trained 60–70 blocks á 50 trials previously) [2] n/s [3] 4 x 48	[1] n/s ^#^ [2] 2 s bl, 3 s active [3] n/s [4] n/s	Not specified/ not reported	n/s
Hinterberger et al. (2005b) ^¤^	[1] Healthy (no diagnose) [2] Range = 21–39 [3] 12	EQ: n/s [1] Cz [2] vEOG [3] Mastoid; n/s	[1] At least 3, plus 2 fMRI-sessions [2] n/s (max 3 days between fMRI-sessions) [3] 5–10 × 49	[1] 1:1:1^#^ [2] 2 s bl, 2.5 s active, 0.5 s reinforcement [3] n/s [4] n/s	Not specified/ not reported	n/s
Hinterberger et al. (2004a), Pham et al. (2005)	[1] Healthy (no diagnose) [2] ^j^ Pham: *M* (*sd*) = 27.7 9.2; Hinterberger: *M* (*sd*) = 28.7 years (11.2) [3] 59 in complete sample (54 in Hinterberger)	EQ: TTD [1] Cz [2] vEOG [3] Mastoid; n/s	[1] 3 [2] Within 10 days, at least 2 days in between [3] 10 x 50	[1] 1:1 [2] 1 s resting phase, 1 s preparatory phase. 3.5 s active, 0.5 s reinforcement. [3] n/s [4] n/s	Participants were paid 8 euro per hour.	n/s

Active, Active /feedback phase; BCI, Brain-Computer-interface; bl, Baseline/ passive phase; EP, Epilepsy; EQ, Equipment; FB, Feedback condition; FB ±, Activation/deactivation trials during feedback condition; fMRI, functional Magnetic Resonance Imaging; hEOG, Horizontal Electro-Oculogram; n/s, Not specified; s, Second; S, Session; TR, Transfer condition; TR ±, Activation/deactivation trials during transfer condition; TTD, Thought Translation Device; vEOG, Vertical Electro-Oculogram.

* Refers to whole sample;

¤ Included session recorded in an fMRI-environment;

# Included neutral (“passive viewing”) as a third condition.

a 30 sessions took 15–49 weeks, including break.

b Study also included a training period of Theta/Beta-NF.

c Wangler et al. (2011) reported both hEOG and vEOG.

d Leins et al. (2007) reported only 5.5s as active and 0.5s as “Reward phase”.

e Based on the initial inclusion criteria.

f No online-correction, the participant was asked not to blink during trials.

g Positioned on back of the neck.

h Each trial also included a task = 15s/trial.

i During one session the participants tried 300 transfer trials.

j Preliminary data in Hinterberger et al. (2004a), Neumann et al. (2004), complete data set in Pham et al. (2005).

### Samples

The majority of studies included ADHD samples (16/34; 47%), healthy and neurotypical subjects (8/34; 24%) or patients with epilepsy (6/34; 18%). Additional single studies concerned autism, Parkinson's disorder, tinnitus, migraine, psychopathy, Tourette syndrome or amyotrophic lateral sclerosis (ALS). The age of study participants ranged from 7 to 71 years, with 17 studies involving children. Except for the studies by Mayer et al. (2012, 2016), all ADHD studies focused on children and adolescents. Within the BCI-context, studies focused either on patients with ALS (9/14; 64%) or on healthy subjects (4/14; 29%). One study included both patients with ALS and healthy subjects. The age of participants in BCI-studies ranged from 18 to 75 years.

Besides two single subject case-studies (Kotchoubey et al., 2002; Milner et al., 2016), the median number of participants in treatment studies was Md = 17 (range: 8–72). Studies with healthy subjects had a median of 9 (range: 9–27). Four SCP training studies had a sample size of at least *N* = 50, all focusing on ADHD (Gevensleben et al., 2009b; Strehl et al., 2017; Minder et al., 2018; Hasslinger et al., 2022).

Concerning studies within the BCI context, 4 were single cases (Kaiser et al., 2002; Neumann et al., 2003; Hinterberger et al., 2005a; Karim et al., 2006), 6 included two to five participants (Birbaumer et al., 2000; Kaiser et al., 2001; Kübler et al., 2001; Neumann and Birbaumer, 2003; Hinterberger et al., 2004b; Neumann et al., 2004), 2 studies included 12–20 participants (Kübler et al., 2004; Hinterberger et al., 2005b), and 1 study had more than 50 participants (Hinterberger et al., 2004a; Pham et al., 2005).

### Technical equipment

Most BCI studies reported the use of the software of the Though Translation Device (TTD). Many studies reported add-on software for further word-processing (Birbaumer et al., 2000; Kübler et al., 2001, 2004; Kaiser et al., 2002; Neumann et al., 2003, 2004), and one study reported using *Descartes* (Karim et al., 2006), an add-on software that enabled web browsing. Early treatment studies used various EEG-amplifiers, although not always reporting the specific equipment nor software used. However, the epilepsy research conducted by Kotchoubey et al. (2001), used EEG-amplifiers from Nihon Kohden (Nihon Kohden Corp., Japan) and software that was based on the same TTD software as used in the BCI studies. Other studies reported the use of commercially non-available systems “Goettinger Feedback” (GoeFi) (Heinrich et al., 2004; Drechsler et al., 2007) and “Self-regulation and Attention Management” (SAM) (Gevensleben et al., 2009b, 2014a,b; Studer et al., 2014), which had been used in studies for ADHD. Thereafter, commercial systems have become available, and systems by neuroConn (neuroConn GmbH, Ilmenau, Germany) have been established as the most used (~50% of all studies), while equipment from Though Technology (Thought Technology Ltd, Montreal) has been used in two studies (Milner et al., 2016; Morales-Quezada et al., 2019). With the exception of retrospective analysis (Heinrich et al., 2020), all articles published since 2015 (18 reports, covering 12 studies), have utilized one these two abovementioned commercially available systems.

### Electrode placement

With the exception of two studies (Konicar et al., 2015, 2021a), where the active electrode site was set at FCz, and one study that focused on left-hemispheric SCPs at C5 (Pulvermüller et al., 2000), all other studies placed their active electrode at the vertex (Cz). The reference was most often placed on a mastoid or earlobe, while the ground was sometimes placed on the forehead (Takahashi et al., 2014; Okumura et al., 2019), neck (Milner et al., 2016) or at AF3 (Kleinnijenhuis et al., 2008; Spronk et al., 2010). Similarly, all BCI studies placed the active electrode at Cz.

Eye-movement correction was reported in almost all treatment studies, with few exceptions (Uhlmann and Fröscher, 2001; Takahashi et al., 2014; Baumeister et al., 2018; Okumura et al., 2019; Krepel et al., 2020). Studies using the NeuroConn systems, and three studies using other systems (Pulvermüller et al., 2000; Doehnert et al., 2008; Wangler et al., 2011) reported the recording of both horizontal and vertical EOG. All other studies reported the use of only vertical EOG for eye-movement corrections. The BCI studies also reported only using vertical EOG.

### Trial length

There was some variation in trial length, both in the baseline phase and the active phase. Most of the studies using the Neuroconn systems, implemented a 2 s baseline phase, followed by an 8 s active phase. However, there were some exceptions where the active phase only lasted for 5.5 s (Albrecht et al., 2017) or 6 s (Christiansen et al., 2014). Studies using the SAM and the GoeFi-system, report 2 s baseline phase and 6 s active phase. Some studies reported trial length of 8 s without clearly specifying the baseline and active phases (Uhlmann and Fröscher, 2001; Kotchoubey et al., 2002; Hinterberger et al., 2003; Strehl et al., 2006b). A baseline phase of 0.5 s and a 7 s active phase was used in two studies (Kleinnijenhuis et al., 2008; Spronk et al., 2010). Overall, trials lasted between 7.5 and 10 s in total.

Only two studies reported shorted trial length, with 2s for the baseline phase and 3 s for the active phase (Siniatchkin et al., 2000a,b). Such trial length is more consistent with BCI-studies, where the total trial length was typically 4–6 s. The baseline phase (or preparatory phase) was also around 2 s, while the feedback phase only lasted 2–4 s. However, in some cases the active phase lasted for up to 5 s (Neumann and Birbaumer, 2003). The shorter trial length in BCI studies was mainly described to enable more decisions per minute, with increased speed being pivotal when using SCP shift as a means of communication.

### Number of sessions

The number of sessions ranged widely from 1 to 35 across the published studies. Eight studies employed multiple training units per session (Heinrich et al., 2004; Drechsler et al., 2007; Gevensleben et al., 2009b, 2014a,b; Studer et al., 2014; Minder et al., 2018; Okumura et al., 2019). When considering training units instead of sessions, the median was 24 units (range: 1–35). When excluding studies focusing on healthy subjects alone, and a single session experiment (Strehl et al., 2006b), the median was 25 units (range: 3–35).

The number of training blocks varied across studies (range: 1–8 blocks/ session), as did the number of trials per block (range: 30–60). Although the exact number of trials per training unit or session was not always specified, more than 67% (23/34) reported 120–160 trials per session.

In the BCI studies, participants were usually trained until they could regulate their SCPs, and the number of sessions was not predetermined. The training volume (i.e., total number of trials) was adapted to the participants attentional and motivational abilities. This also included adjusting the number of trials per block and session. Often a session consisted of 5–20 blocks, and one block of 50–100 trials. For example, Neumann et al. (2004) reported their participants to complete 179 blocks in 31 sessions, and 249 blocks in 23 sessions, with each block consisting of 70 trials, resulting in 404 and 758 trials per session. In BCI studies focusing on healthy or neurotypical participants, training sessions were more limited, ranging from 1 to 5, with 5 to 12 blocks of 50 to 100 trials each.

### Negativation and positivation trial ratio

The ratio between negativation (activation) and positivation (deactivation) trials was usually set at 1:1 in the beginning of training. Toward the later stages of training some studies changed the ratio to 2:3 or up to 1:4, in order to increase the number of deactivation trials (for epilepsy) or activation trials (in case of ADHD). Eight studies did not report or specify the ratio (Kotchoubey et al., 2000, 2002; Pulvermüller et al., 2000; Uhlmann and Fröscher, 2001; Hinterberger et al., 2003; Gevensleben et al., 2014b; Morales-Quezada et al., 2019; Krepel et al., 2020). The direction (activation or deactivation) was usually presented in a pseudorandom order, with the exception of two studies that either only used one modality per block (Siniatchkin et al., 2000a) or that implemented a fixed order for each session (Milner et al., 2016). Most BCI studies did not report the ratio between activation and deactivation trials, although when reported, it was a 1:1 ratio (Kübler et al., 2004; Pham et al., 2005). Four articles, that examined SCP regulation within a functional magnet resonance imaging scanner (Hinterberger et al., 2003, 2004b, 2005b; Strehl et al., 2006b), utilized in addition to the activation and deactivation conditions, a third condition where the participant was instructed to remain cortically neutral. In these cases, the ratio was 1:1:1.

### Transfer trials

Transfer trials (i.e., trials without continuous real-time feedback) were utilized in almost all treatment studies. Five studies did not specify the use of transfer trials (Uhlmann and Fröscher, 2001; Kleinnijenhuis et al., 2008; Spronk et al., 2010; Morales-Quezada et al., 2019; Krepel et al., 2020). Although transfer trials are a common part of SCP-NF, their implementation varied considerable across studies. Eight studies utilized specific transfer-blocks, consisting of 100% transfer trials. Other studies applied of transfer trials pseudo-randomly intermixed with the continuous feedback trials in every training block. They were either set at a steady percentage, or increased in number over sessions (e.g., 20% first week, 40% second week, 50% thereafter). Generally, the BCI-studies did not utilize transfer trials. However, one study aimed at enabling participants to autonomously turn on the Thought Translation Device (Kaiser et al., 2001). This was accomplished by generating a series of SCP shifts without feedback. Another study integrated 300 transfer trials in one session during training of web browsing (Karim et al., 2006).

### Session frequency

The frequency of training sessions varied between studies. While 3 of 4 epilepsy studies implemented daily training sessions, only 4 of 17 ADHD studies implemented daily training, including 2 studies that only used daily training during the first week (Drechsler et al., 2007; Doehnert et al., 2008; Albrecht et al., 2017). The most common frequency was 2–3 sessions per week. Similarly, there were differences in the utilization of breaks. At least one longer break was planned in the training schedule of 10 out of 34 studies.

In the BCI studies with ALS patients, breaks and session frequency was adjusted for the individual participants and specific frequencies are not reported. However, if specified, then multiple sessions per week were reported (Birbaumer et al., 2000; Kübler et al., 2001; Neumann and Birbaumer, 2003; Neumann et al., 2003). Session frequency was also rarely reported in studies with healthy participants (Pham et al., 2005).

### (Trial-)Thresholds

The use of thresholds, i.e., the minimal amplitudinal change needed for a trial to be counted as successful, was reported in 12 out of 34 studies. Five studies reported thresholds of ± 24–50 μV (Fumuro et al., 2013; Takahashi et al., 2014; Albrecht et al., 2017; Minder et al., 2018; Hasslinger et al., 2022). One study implemented different thresholds for activation trials (-15 μV) and for deactivation trials (10 μV) (Uhlmann and Fröscher, 2001), while another study reported to increase thresholds with 5% once participants achieved success rates of ≥70% (Christiansen et al., 2014). Three studies reported the use of individualized thresholds (Kleinnijenhuis et al., 2008; Spronk et al., 2010; Milner et al., 2016) Here, by applying different threshold-levels in an offline analysis of performance-data from a previous session, a threshold-level was estimated, that would generate a projected success rate of 30–33% in upcoming sessions. Another two studies reported the use of thresholds, but without clearly specifying them (Siniatchkin et al., 2000a,b; Okumura et al., 2019).

Three BCI studies, using the Thought Translation Device, reported the use of thresholds. One study used 7 μV (Karim et al., 2006), another 7.7 μV (Neumann et al., 2003), and the third gradually raised the threshold from 5 to 8 μV (Birbaumer et al., 2000). Another five studies (Kübler et al., 2001, 2004; Kaiser et al., 2002; Neumann and Birbaumer, 2003; Neumann et al., 2004), classified trials as invalid and were repeated, if they did not pass a threshold of ± 0.5 μV.

### Reward systems

Concerning studies in BCI-context, use of reward systems was commonly not reported, except in two experimental studies on healthy subjects, which compensated participants with a fixed sum per hour (Kübler et al., 2004; Pham et al., 2005). No reports concerning participants with ALS, discussed the use of reward systems.

Among the clinical studies, 15 reported information on rewards or compensation to participants. Of these, eight concerned ADHD, which all implemented a token system, where points were collected in each session that later could be redeemed for small gifts, such as stickers, small toys etc. However, only half described a performance based system (Strehl et al., 2006a; Gevensleben et al., 2014b; Albrecht et al., 2017; Baumeister et al., 2018). Another two studies indicated that performance may have influenced the rewards, either by awarding points for successful trials without clarifying their relation to tokens (Drechsler et al., 2007) or tokens given for staying attentive (Christiansen et al., 2014), which may have impacted performance. Two ADHD studies stated clearly that their token systems were based on participation and good conduct only (Strehl et al., 2017; Hasslinger et al., 2022). Unfortunately, the operationalization of implemented token systems was mostly sparse across studies. In two other studies, neurotypical children (Siniatchkin et al., 2000b) and those with migraine (Siniatchkin et al., 2000a), also implemented token systems, rewarding the participants with sweets.

Financial compensation for participation per hour, session or for completion, was reported in studies concerning healthy adults (Pulvermüller et al., 2000; Gevensleben et al., 2014a), psychopathic adults (Konicar et al., 2015) and children with ADHD (Hasslinger et al., 2020). Also concerning healthy adults, Kotchoubey et al. (2000), added a financial bonus for every session in which participants attained a differentiation of at least 5 μV, which was bigger than the financial compensation for participating.

Two studies explicitly mentioned not rewarding their participants beyond the reinforcement phase after each successful trial (Mayer et al., 2016; Milner et al., 2016). Mayer et al. (2016), concerning adults with ADHD, discussed that their lack of reward system might have had a negative effect on participants motivation.

### Transfer-promotion/transfer-exercises

Twenty-one studies reported the use of some sort of transfer-promoting exercises, i.e., practicing self-regulation strategies at home for the purpose of aiding the transfer into the participants everyday life. Thirteen (62%) studies concerned ADHD, three (14%) epilepsy, and one each concerned psychopathy, autism, tinnitus, migraine, or healthy/ neurotypical adults.

In the ADHD studies, practicing regulation/strategies was mainly aided by the use of so-called transfer-cards, which was reported in 7 studies. These transfer-cards displayed pictures from the training session. In addition to these cards, two studies also reported the use of a DVD showing transfer trials. Three studies reported that participants were doing homework in the lab or directly after training, whilst applying the regulatory strategies, under supervision of the trainer (Strehl et al., 2006a, 2017; Baumeister et al., 2018). Five studies reported that parents were instructed to help the participants (Drechsler et al., 2007; Gevensleben et al., 2009b, 2014b; Minder et al., 2018; Hasslinger et al., 2020), and four studies reported use of a log for the transfer exercises (Heinrich et al., 2004; Christiansen et al., 2014; Mayer et al., 2016; Albrecht et al., 2017). Transfer exercises were often introduced after having completed half of the planned sessions, and often introduced before a longer training-break. No study reported any data concerning the transfer exercises, e.g., data from logs or feedback from participants or parents.

In studies concerning other conditions than ADHD, transfer exercises were often limited to the training breaks, with one exception [Study 2 in Gevensleben et al. (2014b)]. Four studies reported the use of transfer-cards or dry-runs (Gevensleben et al., 2014b; Konicar et al., 2015, 2021a; Milner et al., 2016). In addition to practicing learned strategies during an 8-week break, as for all three studies concerning epilepsy, Kotchoubey et al. (2001) also reported that every session included a behavior therapy, that “…*was intended to increase patients' awareness of antecedents of seizure behavior, change reinforcing contingencies, and transfer self-control skills to everyday life.”*(Strehl et al., 2005, p. 158). In their study on healthy adults, Studer et al. (2014) implemented an attention demanding task for the last 10 min of the session, where the regulation strategies were practiced.

### Evaluation of self-regulation

Just over half of the articles (32 of 63), reported data concerning regulatory success. Overall, data was reported from 17 clinical studies and 9 BCI-studies.

Within the BCI-context, correct differentiation is more important than correctly increasing or decreasing the amplitude compared to baseline, since participants are meant to employ their SCP regulation as a means for binary communication. Therefore, self-regulation in BCI-studies was primarily evaluated based on the percentage of successful trials. The trial was judged as correct if the generated SCP-shift corresponds with prompted modality, i.e., increase in positivation during deactivation trials and increased negativation during activation trials. Typically, a stable mean success rate of 70–75% was necessary in order to progress to the next word-processing stage. Additionally, some studies implemented thresholds that had to be surpassed in order to be classified as successful (Birbaumer et al., 2000; Neumann et al., 2003; Karim et al., 2006). In other instances, trials were retaken if the amplitudinal changes were too small (Kübler et al., 2001; Kaiser et al., 2002; Neumann and Birbaumer, 2003; Neumann et al., 2004). One study reported re-calculations that adjusted the baseline (i.e., recalibrating the baseline so it was set between the mean activation and deactivation curves), in order to optimize the differentiation between the two polarities and maximize the percentage of correct trials (Pham et al., 2005).

Although five clinical studies also utilized the percentage of correct trials to evaluate self-regulation (Takahashi et al., 2014; Albrecht et al., 2017; Strehl et al., 2017; Baumeister et al., 2018; Okumura et al., 2019), they most often evaluated self-regulation via either the ability to differentiate between the polarities (i.e., mean amplitude of deactivation trials minus mean amplitude of activation trials), or via changes in amplitude for the polarities separately. Differentiation was assessed in 12 articles, and separate amplitudinal polarities in 9 articles, while 10 used multiple measures.

Most studies evaluated the regulatory performance during feedback and transfer trials separately. However, some focused only on transfer trials (Drechsler et al., 2007; Doehnert et al., 2008; Fumuro et al., 2013; Mayer et al., 2016; Baumeister et al., 2018; Hasslinger et al., 2022), based on the assumption that correct “*differentiation between positivation and negativation during the transfer condition is the highest level of self-regulation skill that can be reached*” (Mayer et al., 2016). In two instances, feedback and transfer trials were intermixed (Gevensleben et al., 2014a; Okumura et al., 2019), and in another case only feedback trials were considered (Takahashi et al., 2014).

Thirteen articles used changes over time (i.e., changes between sessions) as measure to evaluate self-regulation, while five focused on the performance at a specific time point, either the second half of training (Drechsler et al., 2007; Doehnert et al., 2008), toward the end of training (Mayer et al., 2016; Hasslinger et al., 2022) or for booster-sessions at follow-up (Strehl et al., 2014).

Many articles did not divide participants into learners or non-learners. However, if they did, categorization was either based on a regression slope, on changes over multiple time points (Strehl et al., 2017; Baumeister et al., 2018; Zuberer et al., 2018; Aggensteiner et al., 2019), or a median-split (Strehl et al., 2006a; Drechsler et al., 2007; Doehnert et al., 2008; Studer et al., 2014). A specific criterion, e.g., having mean amplitude values for both activation and deactivation trials, were rare (Mayer et al., 2016; Hasslinger et al., 2022). Reports that did not specify learners and non-learners, based their evaluation of regulatory performance on the sample as a whole, by statistically testing differences over time or conditions (Hinterberger et al., 2005b; Strehl et al., 2005, 2014; Leins et al., 2007; Gevensleben et al., 2014a,b; Konicar et al., 2015, 2021a; Albrecht et al., 2017). Regulatory data for individual participants was only provided in 4 articles (Doehnert et al., 2008; Mayer et al., 2016; Milner et al., 2016; Hasslinger et al., 2022). Another BCI-studies provided regulatory data for individual participants (Neumann and Birbaumer, 2003; Kübler et al., 2004). For an overview on how the different articles evaluated successful self-regulation, see Table 2.

**Table 2:** Overview of articles that evaluated self-regulation.

**Article**	**Sample: [1] Diagnosis; [2] Age; [3] sample size**	**Measurement: [1] Type; [2] Time window of active phase; [3] Modality;**	**Evaluation of Learners: [1] Evaluated timepoints; [2] Learners Classified by**	**Results: [1] Outcomes (Amplitudes); [2] Percentage of learners**
Doehnert et al. (2008)	[1] ADHD [2] Range = 9–12 [3] 14	[1] Mean amplitude [2] n/s [3] TR-	[1] S7–14 [2] Median split	[1] Mean TR-amplitude: Range = −0.14 to −4.15 μV [Good]; Range = 0.27 to 5.31 μV [Poor] [2] 50% (7 of 14) (Regulatory data for individual participants is provided).
Drechsler et al. (2007)	[1] ADHD [2] Range = 9–12 [3] 17	[1] Differentiation [2] n/s [3] TR	[1] S7–14 [2] Median split	[1] Differentiation: Mean = 2.71 μV (SD = 3.6) [Overall]: Mean = 5.72 μV (SD = 2.5) [Good]; Mean = 0.034 μV (SD = 1.9) [Poor] [2] 47% (8 of 17)
Mayer et al. (2016)	[1] ADHD [2] Range = 18–60 [3] 24	[1] Differentiation [2] Seconds 3–8 (of 8 s) [3] TR	[1] S27–29 [2] (Mean TR+ minus TR-) > 0	[1] Differentiation: Range: 1.25 μV to 38.65 μV; mean: 10.36 μV [Good] Range: −23.35 to −2.24 μV; mean: −9.22 μV [Poor] [2] 46% (11 of 24) (Regulatory data for individual participants is provided).
Studer et al. (2014)	[1] No clinical diagnosis [2] Range = 19–31^*^ [3] 19	[1] Differentiation and mean amplitude [2] n/s [3] TR and FB	[1] S1+2 and S9+10 [2] Median split	[1] [Total (*n* = 17): Pre: Mean = 1.02 μV (SD = 1.43) Post: Mean = −0.20 μV (SD =2.63) Significant difference between good and poor performers: *t*_(15)_ =-1.40, *p* < 0.10, Cohen's *d* = 0.32 [2] n/s
Strehl et al. (2006a)	[1] ADHD [2] Range = 8–13 [3] 23	[1] Mean amplitude [2] n/s [3] TR-	[1] S21–30 [2] Median split	[1] Mean TR- amplitude: −5.27 μV [Good] −0.051 μV [Poor] [2] n/s
Leins et al. (2007)	[1] ADHD [2] Range = 8–13 [3] 19	[1] Differentiation and changes in amplitude over time. All participants. [2] Whole active phase (5.5 s) [3] TR and FB	[1] S2+3, S29+30 and S32+33 [FU] [2] Did not categorize participants (Analyses based on whole sample).	[1] Differentiation increased over time for FB, but not for TR. Differentiation was significant for TR at s.29 + 30 (*p* = 0.036, ES = 0.81) and at s.32 + 33 (*p* = 0.048, ES = 0.90) Amplitude in activation trials (–) increased significantly over time, for both TR and FB. Not for deactivation trials. [2] n/s
Strehl et al. (2005)	[1] Epilepsy [2] Range = 17–50 [3] 34	[1] Differentiation. Also, a “transfer coefficient score” = ratio between differentiation of FB and TR. [2] Seconds 2–8 (of 8 s). [3] n/s	[1] S1–20 and S21–35 + last session; mean for booster sessions [2] Based on seizure reduction (Analyses based on whole sample.)	[1] Categorization only based on seizure reduction. [Mean SCP amplitude, first phase SCP: Improved: 1.63 ± 1.008; Indefinite: −0.32 ± 1.480; Fail: −1.31 ± 1.711. SCP differentiation, last session: Improved: 2.33 ± 1.065; Indefinite: 2.88 ± 0.668; Fail: 1.40 ± 0.783] [2] n/s
Strehl et al. (2014)	[1] Epilepsy [2] Range = 31–59 [3] 16	[1] Differentiation [2] Seconds 5–8 (of 8 s.) [3] FB and TR	[1] Booster sessions at 10-year FU [2] Did not categorize participants (Analyses based on whole sample).	[1] Significant differentiation for FB (p = 0.038) but not for TR (*p* = 0.15). [2] n/s
Fumuro et al. (2013)	[1] Parkinson + healthy controls [2] Parkinson: Range = 36–71 Healthy controls: Range = 60–69 [3] 10 + 11	[1] Differentiation [2] Seconds 4–8 (of 8 s.) [3] TR	[1] Judged each session individually. [2] Judged sessions (instead of participants) as successful if correct differentiation during TR.	[1] n/s [2] Session based. Parkinson: 47% (*n* = 8); Controls: 60% (*n* = 12) Subject based: Parkinson: 4 of 7; Controls: 5 of 9
Konicar et al. (2015)	[1] Psychopathy [2] *M (SD)* = 43.14 (11.52) [3] 14	[1] Differentiation and amplitudes [2] seconds 4-8 (of 8s.) [3] FB and TR	[1] First six sessions and last six sessions. [2] Did not categorize participants. (Analyses based on whole sample.)	[1] Significant learning progress for FB (p =0.048) and TR (p =0.018) Increase in FB- amplitude (p =0.038), FB differentiation [FB+ minus FB-] (p = 0.049) and total regulation [both FB and TR together] (p =0.005). Differentiation: FB: from M = 4.6 μV (SD = 1.32 μV) to 11.6 μV (SD = 7.2 μV); TR: from M = −0.46 μV (SD = 2.3 μV) to M = 5.0 μV (SD = 7.0 μV) [2] n/s
Strehl et al. (2017)	[1] ADHD [2] *M (SD)* = 8.6 (0.92) [3] 72	[1] Mean amplitude and mean PCT [2] Seconds 4–8 (of 8 s) [3] FB and TR	[1] Average for S2+3, S10+11, S14+15 and S23+24. [2] Based on regression slope, separate for FB and TR.	[1] Over all sessions, correct differentiation only in FB, not TR (Data presented graphically). [2] Based on regression slope: FB: 67.9%; TR: 53.7%.
Aggensteiner et al. (2019)	[1] ADHD [2] *M (SD)* = 8.6 (0.92) [3] 72	[1] PCT [2] n/s [3] FB and TR	[1] S2+3, S10+11, S14+15 and S23+24 + FU [2] Based on regression slope	[1] Mean percentage of correct trials = 44% [2] FB = 63.5%; TR = 58.3% in TR
Minder et al. (2018)	[1] ADHD [2] Range = 8–16 [3] 44 (23 in clinic; 21 in school)	[1] Mean amplitude per session [2] seconds 2–8 (of 8 s.) [3] FB and TR	[1] All sessions [2] Successful learning = Negative slope in activation trials, or positive slope in deactivation trials. Successful regulator = Correct slope in both conditions.	[1] (Data presented graphically.) [2] FB-: 20 (41.7%); FB+: 23 (47.9%) FB Regulators: 10 (20.8%) TR-: 23 (47.9%); TR+: 23 (47.9%) TR Regulators: 8 (16.7%)
Albrecht et al. (2017)	[1] ADHD [2] Range = 7–17 [3] 24 (13 at FU)	[1] Mean PCT [2] 24 μV for 2 s during the second half of trial (of 5.5 s.) [3] FB and TR	[1] All sessions [2] Did not categorize participants (Analyses based on whole sample).	[1] FB- started at 16.46% ended at 24.29% (peak 26.64%). TR- started at 18.25% ended at 20.94% (peaked at 24.49%) [Numbers for deactivation trials are not discussed; data presented graphically]. [2] n/s
Okumura et al. (2019)	[1] ADHD [2] Range = 7–16 [3] 22	[1] PCT [2] 2 s consecutively above threshold (not specified). [3] FB and TR combined	[1] First 6 sessions and last 6 sessions. [2] Based on relative improvement during deactivation trials^**^.	[1] (Data presented graphically.) [2] 45.5% (10 of 22), based on positivation trials only
Takahashi et al. (2014)	[1] ADHD [2] Range = 8–16 [3] 10	[1] PCT and peak amplitude [2] 2 s above threshold (± 40μV) [3] FB	[1] Sessions 1-16 [2] Did not categorize participants. (Analyses based on whole sample.)	[1] (Data presented graphically.) [2] n/s
Baumeister et al. (2018)	[1] ADHD [2] Range = 9–14^*^ [3] 8	[1] PCT [2] n/s [3] TR	[1] Slope based on all 20 sessions. [2] Positive slope = learner; Negative slope = non-Learner.	[1] *R*^2^ =0.002, for whole group (Data presented graphically). [2] 50% (4 of 8)
Hasslinger et al. (2020)	[1] ADHD [2] 9–17^*^ [3] 14	[1] Differentiation, mean amplitude [2] Seconds 5–8 (of 8s) [3] TR and FB	[1] Average for last 3 sessions [2] Differentiation with correct direction	[1] (Data presented graphically.) [2] n/s
Hasslinger et al. (2022)	[1] ADHD [2] 9–17^*^ [3] 51	[1] Differentiation, mean amplitude [2] Seconds 5–8 (of 8 s) [3] TR and FB	[1] Average for last 3 sessions [2] Differentiation with correct direction	[1] (Data presented graphically.) [2] 26% (13 of 49) (Regulatory data for individual participants is provided).
Gevensleben et al. (2014b)	*Study 1:* [1] ADHD [2] Range = 10–13 [3] 10 *Study 2:* [1] Tourette syndrome [2] Range = 9–16^*^ [3] 16	*Study 1:* [1] Polarity (amplitude) [2] Seconds 4–8 (of 6 s)^*^ [3] FB (included some TR) *Study 2:* [1] n/s [2] n/s [3] n/s	*Study 1:* [1] S1, S5, S9 and S13 [2] Did not categorize participants (Analyses based on whole sample.) *Study 2:* [1] n/s [2] n/s [3] n/s	*Study 1:* [1] (Data presented graphically.) [2] n/s *Study 2:* [1] n/s [2] n/s
Gevensleben et al. (2014a)	[1] Healthy [2] Range = 18–29^*^ [3] 9	[1] Mean amplitudes and differentiation [2] Seconds 2–6 (of 6 s) [3] FB and TR mixed	[1] Mean for each session [2] Did not categorize participants (Analyses based on whole sample).	[1] (Regulatory data is presented for each session and presented graphically). [2] n/s
Konicar et al. (2021a)	[1] ASD [2] *M (SD)* = 14.05, (1.76) [3] 21	[1] Change in mean amplitude and differentiation [2] Seconds 4–8 (of 8 s.) [3] FB and TR, each block separate.	[1] All sessions [2] Did not categorize participants (Analyses based on whole sample).	[1] (Data presented graphically.) [2] n/s
Milner et al. (2016)	[1] Tinnitus [2] 50 [3] 1	[1] Mean amplitude and differentiation [2] FB and TR	[1] Mean for each session [2] n/s	[1] (Regulatory data is presented for each session and presented graphically). [2] n/s
Birbaumer et al. (2000)	[1] ALS [2] n/s [3] 5 (of which 2 discontinued)	[1] PCT [2] n/s [3] FB	[1] n/s [2] Stable performance of 75%	[1] (Data presented graphically.) [2] 3 of 5 (1 within few weeks, 2 needed several month)
Kaiser et al. (2001)	[1] ALS [2] 43 and 31 [3] 2	[1] PCT [2] Integral of trial (of 3 s) [3] TR	[1] n/s [2] n/s	[1] Patient 1: reached 84.1% Patient 2: reached above 90% [2] n/s
Kübler et al. (2001)	[1] ALS [2] 45 and 31 [3] 2	[1] PCT [2] Average of entire active phase (of 2.5–4 s) [3] FB	[1] n/s [2] Stable performance of 70% (with increasing trend, max performance of at least 75%)	[1] (Data presented graphically.) Stable performance after 82 and 121 blocks, respectively. [2] 2 of 2
Kaiser et al. (2002)	[1] ALS [2] 43 [3] 1	[1] PCT [2] Average of entire active phase (of 3 s) [3] FB	[1] n/s [2] Stable performance of 75%	[1] (Data presented graphically.) Stable performance > 85% after 18 training days [2] 1 of 1
Neumann and Birbaumer (2003)	[1] ALS [2] Range = 31–66 [3] 5	[1] PCT [2] Average of entire active phase (of 3–5 s) [3] FB	[1] Block 1–30; Block 64–93; Block 162–191. [2] PCT of 70%	[1] (Data presented graphically) 70% reached after 86 and 121 blocks respectively. [2] 2 of 5 (Regulatory data for individual participants is provided.)
Kübler et al. (2004)	[1] ALS + healthy (no diagnose) [2] ALS: Range = 35–63 Healthy: Range = 18–39 [3] 10 + 10	[1] PCT, linear and power trends [2] Average of entire active phase (of 4 s) [3] FB	[1] Healthy: within 6 sessions ALS: within 12 sessions [2] Reaching 70% (within 6 or 12 sessions)	[1] (Data presented graphically). [2] Healthy: 8 of 10 ALS: 5 of 10 (Regulatory data for individual participants is provided).
Neumann et al. (2004)	[1] ALS [2] n/s [3] 2	[1] PCT and differentiation [2] Average of entire active phase (of 3s) [3] FB	[1] Participant 1: Completed in 179 blocks; Participant 2: Completed 249 blocks. [2] n/s	[1] (Individual data presented graphically). [2] 2 of 2
Hinterberger et al. (2005b)	[1] Healthy (no diagnose) [2] Range = 21–39 [3] 12	[1] PCT, and effect size of differentiation [2] n/s [3] FB	[1] One session each: SCP-NF, Simulator, fMRI. [2] Did not categorize participants (Analyses based on whole sample).	[1] (Individual data presented graphically). SCP-NF: 66.8%, ES 0.72 Simulator: 64.0%, ES 0.70 fMRI: 69.0%, ES 1.05 [2] n/s
Hinterberger et al. (2004a)	[1] Healthy (no diagnose) [2] Range = 18–75 [3] 54	[1] PCT, effect size of differentiation [2] n/s [3] FB	[1] All three sessions [2] PCT <70%.	[1] (Individual data presented graphically). Differentiation improvement: Visual-FB (*r*^2^ = 0.88, *p* < 0.001); Auditory-FB (*r*^2^ = 0.64, *p* < 0.001); Combined-FB (*r*^2^ = 0.23, *p* < 0.01). [2] PCT >70%: Visual-FB (6 of 18); Auditory-FB (4 of 18); Combined-FB (2 of 18)

ALS, Amyotrophic lateral sclerosis; ES, Effect size: n/s, Not specified; PCT, Percentage of correct trials; S, Session; sd, Standard deviation.

* Based on whole sample.

** Significant difference in mean success rate was not found for negativation trial, upon which only positivation trials were analyzed further.

Another aspect that needs to be considered when evaluating self-regulation, is how the “time window” was set. This determines what segment of each trial was considered for the evaluation. In BCI studies, this time window was based on the trial's entire active phase. A trial was deemed correct if the average amplitude of the active phase was greater or lower (depending on the prompted direction; deactivation or activation trial) than the average amplitude of the baseline phase. The use of the entire active phase was reported in 6 of 9 articles, although the trial length varied between 2.5 and 5 s. The remaining 3 articles did not report any information on this.

In clinical studies the time window commonly varies more than in BCI studies. Only one clinical study utilized the entire active phase (Leins et al., 2007). It was more common to measure the average amplitude of the trial's final 3 s (Strehl et al., 2014; Hasslinger et al., 2020, 2022), 4 s (Fumuro et al., 2013; Gevensleben et al., 2014a,b; Konicar et al., 2015, 2021a), 5 s (Mayer et al., 2016), or 6 s (Strehl et al., 2005; Zuberer et al., 2018). This means that the first 2–5 s of the active phase were not considered, and the SCP signal was given time to “develop.” Three articles reported that trials were deemed successful if the amplitude surpassed a threshold of 2 s. One article specified that these 2 s had be consecutive during the trials second half (Albrecht et al., 2017), another article also stated they had to be consecutive, but without specifying during what period (Okumura et al., 2019), while the third article did not specify whether the 2 s had to be consecutive nor during what period of the trial (Takahashi et al., 2014).

### Outcome of successful self-regulation

Across studies, the terminology of describing successful self-regulation varied. Some used the terms learner and non-learner, while others used regulator and non-regulator. Still others described participants as good or poor performers. Although there are some nuances between the meaning of these terms, in this review we use them interchangeably. There are important differences between studies within the BCI-context and more clinically oriented studies. In BCI studies successful self-regulation is often the main goal, while clinical studies have a greater interest in symptom reduction. Therefore, many BCI-studies kept on until a rate of 70–75% correct trials were achieved. Contrary, clinical studies always implemented a fixed number of sessions in advance. The rate of reported successful participants is therefore high in BCI-studies, without being very comparable with the outcomes of clinical studies.

The majority of clinical studies, reporting on self-regulatory outcomes, concerned ADHD (12 of 17). Other studies covered epilepsy (Strehl et al., 2005, 2014), psychopathy (Konicar et al., 2015, 2021b), autism (Konicar et al., 2021a), Parkinson's disorder and healthy adults (Fumuro et al., 2013), or healthy adults only (Gevensleben et al., 2014a).

Only seven studies reported the number of individuals that were classified as learners. All studies concerned ADHD. Two articles reporting on the same sample (Strehl et al., 2017; Aggensteiner et al., 2019), classified 63.5% and 67.9% as learners for the feedback condition, and 58.3 and 53.7% for the transfer condition. Classification was based on the regression slope of differentiation over four time points, plus the booster-sessions during follow-up (Aggensteiner et al., 2019). However, no other studies reported learner-rates above 50%. Mayer et al. (2016) reported a similar result, with 46% (11 of 24) classified as learners, while only classifying those that achieve correct differentiation (mean deactivation μV minus mean activation μV = above zero μV) during transfer trials of sessions 27–29. Implementing a stricter classification, Hasslinger et al. (2022) only classified those that had both a negative mean μV during activation trials, and a positive mean μV during deactivation trials, concerning transfer trials of the three last sessions. Only 26% (13 of 49) achieved this. Baumeister et al. (2018) based their classification on an increase of the percentage of correct trial, over all sessions. Half (4 of 8) participants managed a positive slope and were classified as learners. Zuberer et al. (2018) also utilized the slope over all sessions, however, they focused on changes in amplitude. Successful learners were defined by a positive slope for deactivation trials, and a negative slope for activation trials. Individuals that achieved both goals were classified as regulators. For the transfer condition, 47.9% (23 of 48) were classified as learners for activation, and 47.9% (23 of 48) for deactivation trial. However, only 16.7% (8 of 48) were classified as regulators. Results were similar for the feedback condition (activation 47.9%; deactivation 41.7%; regulators 20.8%). Okumura et al. (2019) based their classification on the relative improvement from the first 6 sessions relative to the last 6 sessions, and found 45.5% (10 of 22) learners. Contrary to the other reports, this classification was only based on deactivation trials of both feedback and transfer trials combined, significant differences (on group level) were only found for these.

Another common approach was to separate participants' performance via a median split. Strehl et al. (2006a) separated their participants based on the mean amplitude during transfer activation trials, with a difference between successful regulators (−5.27 μV) and unsuccessful regulators (−0.051 μV). Similarly, Studer et al. (2014) found differences between their good and poor performers, concerning the ability to produce negativity during activation trials. Based on the second half of training, Drechsler et al. (2007) found that their good performers had a differentiation of 5.72 μV, compared to 0.034 μV for the poor performers, during the transfer condition. Based on the same sample, Doehnert et al. (2008) only evaluated the mean amplitude during transfer activation trials, finding that half (7 of 14) had a mean amplitude corresponding correctly (−0.14 to −4.15 μV).

Reporting self-regulation data on a group level was common. Four articles reported data on the mean percentage of correct trials. Albrecht et al. (2017), who utilized a threshold of 24 μV that had to be surpassed for a successful trial, reported a peak success rate of 26.64% for the feedback condition and a peak rate of 24.49% for the transfer condition. Baumeister et al. (2018) reported a mean percentage of success rate of 46.52%, without specifying the implementation of any thresholds. Another common approach was to evaluate performance based on a differentiation (mean deactivation μV minus mean activation μV).

Leins et al. (2007) reported increased differentiation over time, only for the feedback condition but not for the transfer condition. However, they did find significant differentiation for the transfer condition toward the end of training and at follow-up. This was mainly due to an amplitude increase in activation trials, but not during deactivation trials. Similarly (Gevensleben et al., 2014b) found that their participants achieved negative mean amplitudes in activation trials, but not positive mean amplitudes in deactivation trials. In an experiment with healthy adults, the same authors found likewise improved differentiation based on improved regulation during activation trials (Gevensleben et al., 2014a).

Instead of evaluating the individual participants performance, Fumuro et al. (2013) classified each session as good or poor. They found that their participants with Parkinson's disorder had 47% good performance sessions, compared to 60% for their healthy controls. Evaluating performance based on the sample as a whole was also common. When studying epilepsy, Strehl et al. (2005) focused their evaluation of self-regulation on its relation to seizure reduction. However, they found significant differentiation ability for the feedback condition but not for the transfer condition during their 10-year follow-up (Strehl et al., 2014).

Konicar et al. (2015) evaluated their psychopathic participants' performance, found significant improvements differentiation over all sessions, for both the feedback and the transfer condition. However, when comparing the first six sessions with the last six sessions, improvement was only found for the feedback condition but not the transfer condition. In their study on autism, Konicar et al. (2021a) evaluated self-regulation for each block separately. Using a quadratic model, they only found improved differentiation for the first feedback block. No clear patterns were found for the transfer block, nor the second feedback block.

One study compared visual-feedback, auditory-feedback or a combination of both (Pham et al., 2005). Participants were trained for three sessions and had to reach >70% correct trials. Results indicated that the visual-feedback-only group had the highest rate of success (6 of 18), the combined group the lowest rate (2 of 18) and the auditory-feedback-only group performed in between (4 of 18).

### Quality appraisal

Our appraisal indicates an increase in scores of pre-experimental factors (item 1) since 2014. Concerning control groups (item 2), scores were generally low regarding blinded measures. Similarly, control measure (item 3) scores were stable over time. However, psychosocial factors and the reporting of strategies were considerably rare. Contrarily, feedback specifications (item 4) scores were generally high and stable over time. Both the outcome measures brain (item 5) and outcome measures behavior (item 6) scores were heterogeneous. The data storage criteria (item 7) was only fulfilled by one article. When considering studies with multiple articles, the aggregated scores indicate a slight trend toward higher scores over time. The scores per individual article as well as for the studies as a whole (aggregations of multiple articles), are presented in Supplementary Tables S3a–S3c.

## Discussion

This review sought to examine SCP-NF study protocols and variations in the evaluation of self-regulation in SCP-NF studies to aid future standardization and possibly optimization of protocol designs and evaluation methods, especially in clinical application. Sixty-three articles from the past 20 years were included utilizing SCP both within a BCI-context and NF in clinical settings. Arns et al. (2014) introduced the term of “standard protocols”, describing protocols that were well-researched and studied over four decades. Although such a term may indicate that these protocols are comparable or standardized, this review showed that at least for SCP-NF this is not the case. Studies examined here, varied not only in the extent and intensity of training, as well as differences in protocol details (e.g., trial length, thresholds, ratio of polarities), but also in the promotion of self-regulation skill-acquisition, and the methodology of evaluating regulatory ability.

Based on the totality of protocol variations, it is fair to assume that comparability between studies and findings may be substantially hindered. One of these variations concerned the number of sessions. Here, comparability was mainly curbed by the wide spread of trials conducted during each session. Studies that reported a similar number of sessions could differ considerably on the basis of the total number of trials completed. Since the number of trials per block and number of blocks per training session fluctuate, it may be more reasonable to report the total number of trials, in order to increase comparability between future studies. However, as many studies use the same (commercial) systems, certain aspects of protocol-details may become more uniform. For example, online eye-movement corrections using both horizontal and vertical EOG have become more common. Also, the length of each trial is most often set at 10 s (2 s baseline phase and 8 s active phase). Although some use a 6 s active phase, in the past trial length varied considerably more. However, as such protocol-details are not justified theoretically, this may be due to the use of default settings of the systems. Furthermore, settings that are easily adjusted, like the ratio of polarities, the use of thresholds, as well as how transfer trials are implemented, still vary considerably.

Successful trials in SCP-NF were usually rewarded in some way. Most often an animation was displayed on screen. Yet, such simple reinforcers may quickly become dull with limiting motivating effects. Therefore, additional token-systems to further incentivize correct regulation, were frequently utilized in studies reviewed in this article. However, these token-systems were not always connected to the participants' regulatory performance. Sometimes mere participation and good conduct were rewarded or compensated. Overall, token-systems were not well-described in the reports, leaving uncertainty about what exactly is being rewarded. More importantly, no study that implemented a performance-based system, reported on its outcomes, i.e., neither mean and range of tokens rewarded, nor any analysis thereof. Therefore, it is unclear if the tokens have any effect on performance or other outcome measures, including impact on the participants cooperation and motivation.

In recent SCP-NF studies, transfer exercises have become an essential part of training, in particular when concerning ADHD. Most commonly reported were the use of so-called transfer cards, and videos from transfer trials. Both these materials served as visual aids, to help the participants to simulate the training and practice the participants strategies to self-regulate one's SCPs, and were introduced either during a training break, or after a few weeks of training. Usually, it is reported that participants (and their parents) were instructed to use these aids and practice self-regulation, on a daily-basis, in relevant every-day situations. Although, some report the use of logs, no data from the logs is being reported. Similarly, information concerning strategies and the every-day situations, is very sparse. Some single studies report that the participants were assisted to find regulation strategies, and to identify relevant every-day situation, unfortunately without specifying these. These limitations make it difficult to evaluate adherence and quality of these components. Another limitation is that the self-regulatory ability is hardly determined before transfer exercises are being implemented. Many participants may not be able to find stable strategies for successful regulation (Hasslinger et al., 2020), at least within limited time. Hence, one may question the usefulness of practicing strategies that the participant is not fully aware of, as it may increase confusion and negatively impact on motivation.

Concerning evaluating SCP self-regulation, two main approaches were identified. One focuses on the regulatory *ability* at a specific moment, most often toward the later training sessions. Participants can either be categorized (i.e., as regulators or non-regulators) based on an absolute performance criterion (e.g., Hasslinger et al., 2022) or relative performance criteria like a median-split (e.g. Drechsler et al., 2007). While this approach emphasizes the participant's regulatory capacity in a given moment, the other approach focuses on *progression*. Here, the participants' relative improvements over time are considered, comparing the average of the early sessions to latter sessions, or all sessions are used to calculate a slope, upon which direction the “regulator” is defined. This means, that someone who performs well from the beginning and maintains their performance toward the end, would be classified as “regulator” with an *ability* approach, but as a “non-regulator” when using the *progression* approach. Similarly, someone starting with a limited regulatory capability during the initial sessions, but improves toward the end of training (even with limited improvements) may be classified as “regulator” via a *progression* approach, but not via an *ability* approach.

In addition, two different measures were utilized independently of the approach. Common in BCI studies were evaluations based on the *success rate*, i.e., the percentage of successful trials. A trial is deemed successful if the signal is shifted in the same direction as prompted for the trial. In some cases, thresholds were implemented, in order to limit incongruent responses influenced by arbitrary amplitude shifts. Since most BCI-studies aim to utilize SCP-regulation as a way for binary communication, focusing on success rate may cohere better than emphasizing a measure based on *amplitudinal change*. This was more common in the clinical studies. Instead of simply determining whether a trial was successful, the amplitudinal intensity is considered. This may play an essential role for specific symptoms, where the ability to increase (e.g., in ADHD) or decrease (e.g., in epilepsy) cortical excitability is important.

Furthermore, amplitudinal changes were evaluated in different ways. Some evaluated each modality separately (activation/deactivation in feedback/transfer trials) based on *specific improvements*, mostly as part of a progression over time (e.g., an increased amplitude for deactivation trials). Other focus on the *differentiation* ability, i.e., the difference in amplitude between activation and deactivation trials, which can be used for both the performance approach (e.g., categorizing participants based on their differentiation at the end of training) or for the progression approach (e.g., evaluating the difference in differentiation from early sessions to later sessions). In addition, some studies also apply the criterion that regulation must be on the *correct-side*, i.e., negativation trials need a negative value and the positivation trial average needs a positive value. Similarly, for studies using a progression approach, the criterion may be that both activation and deactivation trials must improve over time.

**Figure 2:**
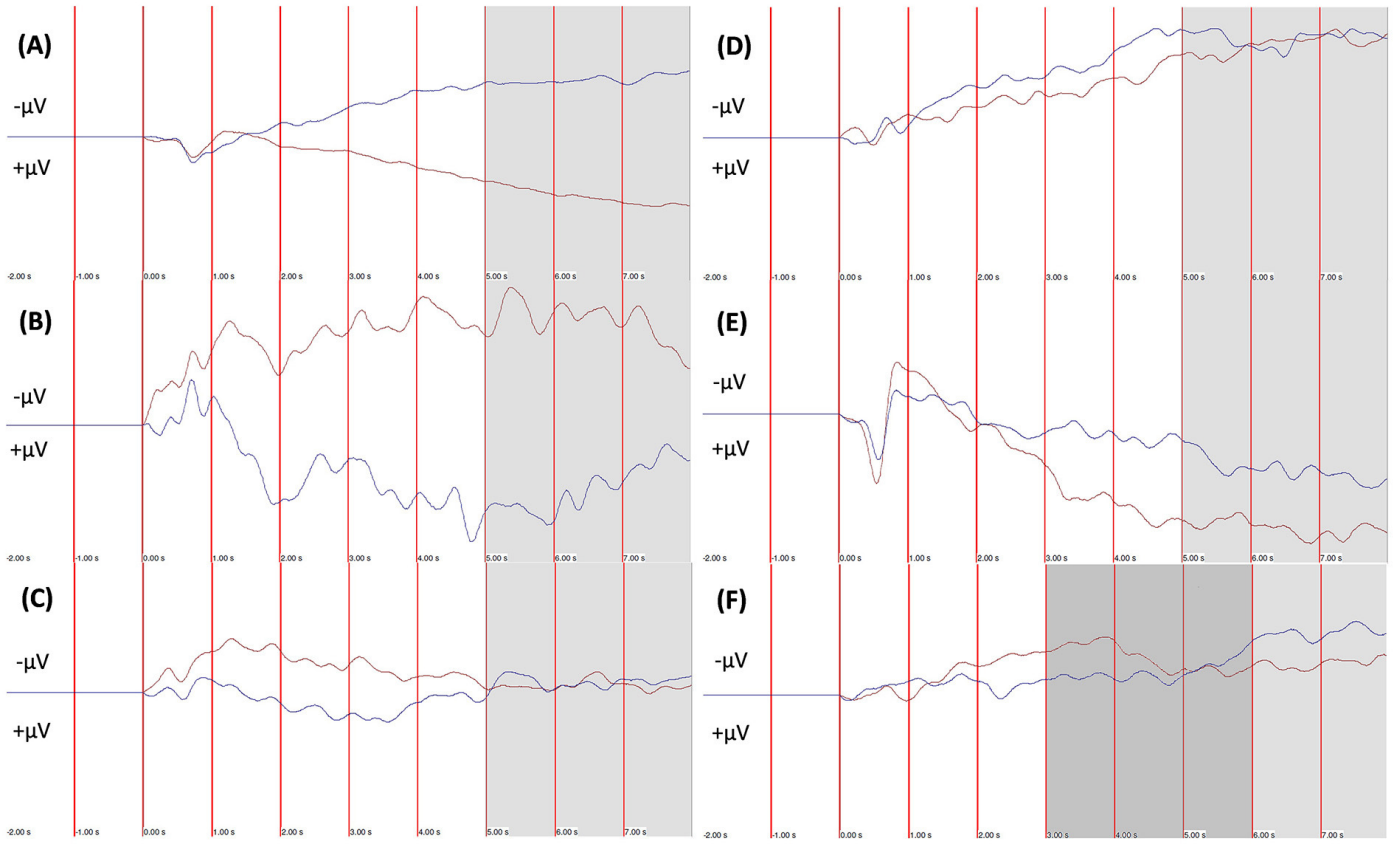
Visualization of the variation of SCP-nf outcomes. The red-line illustrates mean amplitude for all positivation (deactivation) trials, the blue-line illustrates mean amplitude of all negativation (activation) trials. The gray-area indicates last 3 seconds of active phase (5–8 s). **(A)** Profile of a “regulator,” with both negativation and positivation on correct side in relation to baseline. **(B)** Profile of a “inverted-regulator.” There is a clear differentiation between activation and deactivation, but in the opposite direction as instructed. **(C)** Profile of a “non-regulator.” There are barely any differences between activation and deactivation, as both are fluctuating around the baseline. **(D)** Profile of successful deactivation, and differentiation. Althou, activation trials have a lower amplitude than the deactivation trials, their mean is not negative (i.e., wrong side of baseline). **(E)** Profile of successful activation. However, the deactivation trials mimic the activation trials, hence there is merely any differentiation. **(F)** Profile of successful activation and differentiation based on the time measure 5–8 s. However, based on the time measure 3–6 s (dark gray area), as used in most studies not using the NeuroConn system, the profile would show an inverted-differentiation at best.

To illustrate how different evaluation approaches may influence the classification of performance, Figure 2 shows the SCP signal from single sessions from participants from Hasslinger et al. (2020, 2022). The different graphs (A-F) illustrate six different patterns of SCP regulation. Pattern (A) illustrates the optimal outcome, and should always be classified as “regulator.” It shows distinct differentiation between activation and deactivation, with both modalities being clearly above (activation) and below (deactivation) the baseline value, indicating a high success rate. Contrary, although (B) shows a similar pattern of differentiation (as there are clear differences between the modalities), the directions are inverted (i.e., activation goes down and deactivation goes up). Since differentiation mostly is calculated by subtracting the mean activation amplitude from the mean deactivation amplitude, this pattern will not be classified as a regulator, and the success rate be very low. However, there is clear differentiation going on, hence we call this pattern a “inverted-regulator.” There is no clear regulation visible in (C), nonetheless, there is a small difference between the mean deactivation and activation amplitude. Hence, this pattern could be classified as “regulator” both based on the differentiation, and on correct regulation of the activation modality, especially if median-split would be utilized. The success rate would probably be low, particularly if a threshold would be implemented. Similarly, the differentiation in (D) is limited. There is a distinct increase in activation, and the success rate for activation trials would be high, however, there would not have been many successful deactivation trials. There is a clear differentiation in (E), although there is a general deactivation. Hence, if focusing on activation, this pattern would not be classified as “regulator.” Finally, we want to illustrate the potential impact that the chosen time-segment, within which the mean amplitudes are calculated, may have on the classification. If the mean amplitude is calculated for the last 3s (seconds 5–8 when using the NeuroConn systems), then (F) has a positive differentiation, and increased activation. However, if the trials had only lasted 6 s (seconds 3–6, illustrated by the darker gray area), as in 9 studies (see Table 1), then the differentiation would be inverted. Likewise, the classification in (C) would be altered when considering the earlier time-segment. Similarly, the differentiation amplitude in (A) would be considerably smaller. This may limit the comparability between studies with different trial lengths.

Differences in trial length may also influence the training and conditioning itself. In the BCI studies, shorter trials were implemented, in order to increase the number of decisions that could be made. Often these trials were no longer than 4 s, requiring that shifts were initiated soon after the trial started. In more recent clinical studies, trial length is commonly 8 s. This may potentially fatigue the participants more, and have an effect on their motivation. Nonetheless, it may also be that the greatest benefits come from the prolonged generation of activation (or deactivation). Hence, this needs to be considered when evaluating successful regulation. In epilepsy for example, the suppression of cortical excitability is important for seizure reduction, hence the ability to generate positivation shifts (deactivation) may be most beneficial and should be evaluated continuous. To the contrary, in ADHD, the opposite may be more relevant. Although, there are less explicit outcomes noted, it is assumed that an increase in cortical excitability is desired. Hence, many studies increase the number of activation trials compared to deactivation trials. While it is suggested that negativation may aid attention craving tasks, e.g., during school-/homework, it remains unclear whether the ability to generate increased excitability is more important than correct differentiation. The importance of proper differentiation (i.e., on the correct side in relation to the baseline) also remains unclear. Considering that the increasing or decreasing of negativation or positivation always is in relation to the baseline, one may question whether skewed differentiation patterns (i.e. not centered around the baseline) are to be judged differently. Within the BCI-context, some studies have simply “re-centered” the baseline in order to maximize the percentage of correct responses (Hinterberger et al., 2004a; Pham et al., 2005). Such adjustments can be pivotal when SCP-regulation is utilized as a means for communication, as for the TTD. However, there may also be benefits in the clinical context. Progression can easily be tracked in terms of a success rate, which in addition can be linked performance-based rewards. Given the above, future research should evaluate self-regulation on disorder specific measures. However, for comparability, general measure such as differentiation of a percentage of correct trials should also be reported.

Early studies on epilepsy included parallelly implemented behavioral therapy, which aimed to (*1) increasing the subject's perceptual sensitivity to early signs of seizures, as well as to their immediate antecedents; (2) preventing potential seizure triggers and revealing reinforcing contingencies; (3) overcoming possible frustration after intermittent failures on the early stages of biofeedback learning and (4) transfer of the self-regulation skills from the laboratory to everyday life conditions* (Kotchoubey et al., 1996, p. 271). Unfortunately, the approach of merging SCP-NF and behavioral therapy has waned. Some studies focusing on ADHD, introduced their transfer exercises in the “lab” (Strehl et al., 2006a; Drechsler et al., 2007; Leins et al., 2007; Doehnert et al., 2008). However, most current transfer exercises are based on instructions, utilizing transfer-cards or video-clips of transfer-trials, that participants are to conduct at home. A more holistic approach should be sought, including assistance in managing the frustrations due to failing at self-regulation in SCP-NF. Since the regulation of SCPs is very abstract and perhaps even more so for children with ADHD, an overarching and context providing behavioral therapy, could help facilitate benefits from SCP-NF. The participant should learn to identify relevant situations where increased cortical excitability (e.g., during “boring” tasks) and suppressed cortical excitability could be beneficial (e.g., when getting over excited or at bedtime). Furthermore, the transfer exercises should be initiated and trained explicitly within the NF training setting. Also, having all trials in a pseudo-random order may not be optimal for everyone. Studies that examined SCP via fMRI, also utilized neutral-trials in addition to the activation- and deactivation-trials. Such trials may be beneficial for self-regulation for some. Sufficient self-regulatory skills, also seem pivotal when implementing transfer exercises, as the participants need to know what and how to practice their self-regulation skills or strategies. Rather than relying on the participant to learn self-regulation, future SCP-NF studies need to incorporate elements of teaching self-regulation, e.g., by assisting in identify regulation strategies.

Moreover, self-regulation is not necessarily learned linearly. Some participants may show good regulation in the beginning, but seemingly “worsen” over time, some have a steady progression, while others plateau early. There may even be varying trends for different blocks within the same session (Konicar et al., 2021a). Motivation, compliance and the participants training profile/style during training may play an important role (Hasslinger et al., 2020). Especially considering that SCP-NF is very repetitive and monotone. Once the initial novelty factor has worn of, SCP-NF can be extremely boring, and needs addressing. Intended as a distractor, Rockstroh et al. (1993) added a radio program to their transfer modality. Unfortunately, whether this impacted performance is not elaborated. However, to some such “distractors” may make the training more bearable and help with motivation. Furthermore, specific background music could perhaps function as anchor, that could be utilized during transfer exercises outside of the lab/clinic, increasing regulatory transfer. Motivation may also benefit from accentuated assessments point. Rather than considering every training session when evaluating self-regulation, specific assessment sessions or blocks may provide a more realistic picture of the participants capabilities. Similarly, an athlete's progress and abilities are not based on their average performance during all their practices. Rewards could be concentrated to these sessions, allowing for more incentivising values.

Applying the CRED-nf checklist to the selected studies, revealed that SCP-NF is generally well described in its implementation (i.e., data-extraction, set-up, etc.). However, these items may neglect to capture salient components, such as trial length and ratios of transfer-trials, i.e., items that are reported in Tables 1, 2. Also, components that concern the acquisition of self-regulation (i.e., transfer exercises, establishing regulation strategies, etc.) are critical to SCP-NF and should be reported. We therefore suggest that future updates of the CRED-nf should incorporate protocol specific items or addendums, that capture the unique intricacies of the protocol (see Supplementary Tables S3a–S3c).

As this review focused the technical details of the SCP-NF training protocols, predictors for successful self-regulation were not included. Future studies should evaluate biomarkers (e.g., CNV or individual alpha peak frequency) and other predictors, both for symptom improvements and self-regulation success.

## Limitations

Although we utilized search terms as broad as possible, minimizing the risk of missing important contributions, we cannot be certain that we did cover all relevant publications.

The aim of this review was broad and included a multitude of SCP-NF protocol aspects, although the main focus concerned the evaluation of self-regulation. Consequential, a high volume of articles was found and included. Combined with broad extraction targets, this increased the risk of overlooking relevant data.

Also, by not assessing the quality of the included studies prior inclusion, unnecessary low-quality data may have been included, diluting the results. Furthermore, the deficient reporting on aspects concerning self-regulation, may also limited the results of this review.

We did not extract nor report data concerning trials' reward-period, nor data concerning the inter-trial-intervals. This type of data was rarely reported, especially among the clinical studies. For example, Leins et al. (2007) included a 500-msec. reward-period and a 5.5 s active phase. Contrarily, the article by Strehl et al. (2006a), based on the same study, only reported an active phase of 6 s. Hence, some uncertainty concerning the actual trial length exists.

Finally, we did not contact the authors of the included studies to allow them to clarify nor correct potential misinterpretation of their work in this review.

## Conclusions

This review found heterogeneity among SCP-NF training schemes, even for so called “standard protocols.” Differences in trial length, the total training volume, and how performance is evaluated, may skew comparability across studies. However, with an increasing use of commercial systems, there seems to be a trend toward more standardized protocol parameters. Contrarily, the methods to evaluate performance are abundant. Since there is no standard for such evaluation yet, increased reporting on multiple measure is encouraged. On the basis that SCP-NF resonates best within a skill-acquisition framework, future studies need to put more emphasis on the self-regulation ability. Particularly, there is a need to widen the understanding of the mechanisms of how the specific regulatory components interact with different disorders and their symptomatology. Obtaining a sufficient regulatory ability, as well as identifying and applying this ability in relevant everyday-situation, is the essence of SCP-NF. Neglecting these components, may considerably dilute symptom measure outcomes.

## Data availability statement

The original contributions presented in the study are included in the article/Supplementary material, further inquiries can be directed to the corresponding authors.

## Author contributions

JH: conception and design of project, conducted search, screening and selection of studies, data extraction and analysis, interpretation and synthesis, and main contributor to drafting of manuscript. MM: screening and selection of studies, data extraction and analysis, interpretation and synthesis, and drafting of manuscript. SB: principal investigator, overall supervision, funding, editing, and critical revision of the manuscript draft. All authors are contributed to the article and approved the submitted version.

## Funding

This study was funded by Region Stockholm & ALF PPG (Grant Nos: LS2015-1199, HSNV 11590, and HSN 0904-0396). The funders had no role in study design, data collection and analysis, decision to publish, or preparation of the manuscript.

## Conflict of interest

The authors declare that the research was conducted in the absence of any commercial or financial relationships that could be construed as a potential conflict of interest. Additionally, SB discloses that he has in the last 3 years acted as an author, consultant or lecturer for Medice and Roche. He receives royalties for textbooks and diagnostic tools from Hogrefe, and Liber. SB is shareholder in SB Education/Psychological Consulting AB and NeuroSupportSolutions International AB.

## Publisher's note

All claims expressed in this article are solely those of the authors and do not necessarily represent those of their affiliated organizations, or those of the publisher, the editors and the reviewers. Any product that may be evaluated in this article, or claim that may be made by its manufacturer, is not guaranteed or endorsed by the publisher.
