# Supplementary material for: How standardized are “standard protocols”? Variations in protocol and performance evaluation for slow cortical potential neurofeedback: A systematic review
Source: Front Hum Neurosci. 2022 Sep 2;16:887504. doi: 10.3389/fnhum.2022.887504 (PMC9478392; doi:10.3389/fnhum.2022.887504)
Supplement: Supplementary file 1 [file Data_Sheet_1.docx]

**Supplementary Material**

| **Supplementary Table S1 – Documentation of search strategies** |
| --- |
| Documentation of search strategies  University Library search consultation group  Date: May 28 2021  Topic/research question: Vi vill kartlägga hur man definerar deltagare som har lärt sig metoden Slow Cortical Potential (SCP) träning, alltså vem är en "learner" av "successful self-regulation".  Name of researcher(s): John Hasslinger, Institutionen för kvinnors och barns hälsa.  Librarian(s): Anja Vikingson & Jonas Pettersson  Databases:   1. Medline (Ovid) 2. Web of Science (Clarivate) 3. PsycInfo (Ovid) 4. ERIC (ProQuest)   Total number of hits:   - Before deduplication: 1,275 - After deduplication: 800 |
| 1. Medline   \| Interface: Ovid MEDLINE(R) and Epub Ahead of Print, In-Process & Other Non-Indexed Citations and Daily  Date of Search: May 24, 2021  Number of hits:319  Comment: In Ovid, two or more words are automatically searched as phrases; i.e. no quotation marks are needed \| Field labels   - exp/ = exploded MeSH term - / = non exploded MeSH term - .ti,ab,kf. = title, abstract and author keywords - adjx = within x words, regardless of order - * = truncation of word for alternate endings \| \| --- \| --- \| \| **1 Resource selected** \| [Hide](https://ovidsp-dc2-ovid-com.proxy.kib.ki.se/ovid-a/ovidweb.cgi?&S=IGMDFPPDHEEBEFDMJPPJNGBHCBBHAA00&Display+Mode=ovidclassic&Get+Resources+Widget=0) \| [Change](https://ovidsp-dc2-ovid-com.proxy.kib.ki.se/ovid-a/ovidweb.cgi?&S=IGMDFPPDHEEBEFDMJPPJNGBHCBBHAA00&Change+Database=1)   \| [[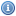](https://ovidsp-dc2-ovid-com.proxy.kib.ki.se/ovid-a/ovidweb.cgi?&S=IGMDFPPDHEEBEFDMJPPJNGBHCBBHAA00&Database+Field+Guide=8)](https://ovidsp-dc2-ovid-com.proxy.kib.ki.se/ovid-a/ovidweb.cgi?&S=IGMDFPPDHEEBEFDMJPPJNGBHCBBHAA00&Database+Field+Guide=8)**Ovid MEDLINE(R) and Epub Ahead of Print, In-Process, In-Data-Review & Other Non-Indexed Citations and Daily**1946 to May 21, 2021 \| \| \| \| \| \| --- \| --- \| --- \| --- \| --- \| \| **#** \| **Query** \| **Results from 24 May 2021** \| \| 1 \| slow cortical potential*.ti,ab,kf. \| 318 \| \| 2 \| scp.ti,ab,kf. \| 3,076 \| \| 3 \| exp Neurofeedback/ \| 1,038 \| \| 4 \| (scp adj3 (neurofeedback or biofeedback)).ti,ab,kf. \| 18 \| \| 5 \| 2 and 3 \| 21 \| \| 6 \| 1 or 4 or 5 \| 319 \| \| \| |
| 2. Web of Science Core Collection   \| Interface: Clarivate Analytics  Date of Search: May 21 2021  Number of hits: 683 \| Field labels   - TS/Topic = title, abstract, author keywords and Keywords Plus - NEAR/x = within x words, regardless of order - * = truncation of word for alternate endings   Note: sometimes “quotation marks” are needed for single search terms to avoid automatic term mapping (lemmatization). \| \| --- \| --- \| \| \| #1 \| TS=("slow cortical potential*" OR "cortical slow potential*") \| 681 \| \| --- \| --- \| --- \| \| #2 \| TS=("SCP" NEAR/2 ("neurofeedback" OR "biofeedback") ) \| 18 \| \| #3 \| #2 OR #1 \| 683 \| \| \| |
| 3. Psycinfo   \| Interface: Ovid  Date of Search: June 17 2021  Number of hits: 270  Comment: In Ovid, two or more words are automatically searched as phrases; i.e. no quotation marks are needed \| Field labels   - exp/ = exploded controlled term - / = non exploded controlled term - .ti,ab,id. = title, abstract and author keywords - adjx = within x words, regardless of order - * = truncation of word for alternate endings \| \| --- \| --- \| \| \| **#** \| **Query** \| **Results from 17 June 2021** \| \| --- \| --- \| --- \| \| 1 \| slow cortical potential*.ti,ab,id. \| 246 \| \| 2 \| (scp adj3 (neurofeedback or biofeedback)).ti,ab,id. \| 12 \| \| 3 \| cortical slow potential*.ti,ab,id. \| 25 \| \| 4 \| 1 or 2 or 3 \| 270 \| \| \| |
| 4. ERIC   \| Interface: ProQuest  Date of Search: May 27, 2021  Number of hits: 3 \|  \| \| --- \| --- \| \| (("slow cortical potential*" OR "cortical slow potential*") OR ((biofeedback AND scp) OR (neurofeedback AND scp)))  3 träffar \| \| |
|  |

**Supplementary Table S2.** Summary of eligible articles published prior year 2000.

| **Author** | **Year** | **Participants** | **Number of participants** | **Age (years)** |
| --- | --- | --- | --- | --- |
| (Kübler et al., 1998) | 1998 | ALS, stroke | 2 | 26 and 41 |
| (Birbaumer et al., 1999) | 1999 | ALS | 2 | n/s |
| (Kübler et al., 1999) | 1999 | ALS, healthy | 3 and 13 | ALS: 50, 37 and 42.  Healthy: 22-54. |
|  |  |  |  |  |
| (Birbaumer et al., 1981) | 1981 | Student volunteers, patients with frontal lobe lesions and normal subjects | 22, 17 and 8 (three experiments) | Adults |
| (Birbaumer et al., 1988) | 1988 | Student volunteers | 34, 14 and 12 (three experiments) | Adults |
| (Birbaumer et al., 1992) | 1992 | Student volunteers | 18 | Range: 20-35 |
| (Brody et al., 1994) | 1994 | Student volunteers | 10 | M = 24.7, SD = 3.6 (range: 19-29) |
| (Elbert et al., 1980) | 1980 | Student volunteers | 17 | 20-24 |
| (Hardman et al., 1997) | 1997 | Student volunteers | 16 | Adults |
| (Holzapfel et al., 1998) | 1998 | Epilepsy, intellectual disability, brain lesion | 1 | 27 |
| (Kotchoubey et al., 1997a) | 1997 | Epilepsy | 18 | M = 33.5 SD = 1.92 |
| (Kotchoubey et al., 1999a) | 1999 | Epilepsy | 34 | M = 34.3 SD = 8.44 |
| (Kotchoubey et al., 1997b) | 1997 | Volunteers | 13 and 5 (two experiments) | Range: 22-54 and 16-31 |
| (Kotchoubey et al., 1996) | 1996 | Epilepsy | 12 | M = 32.2, SD = 2.4 (range: 21-44) |
| (Kotchoubey et al., 1999b) | 1999 | Epilepsy | 28 | M = 32.4 (range: 21-45) |
| (Lutzenberger et al., 1980) | 1980 | Patients with frontal lobe lesions and normal subjects | 25 (8 + 17) | Range: 20-40 |
| (Lutzenberger et al., 1979) | 1979 | Student volunteers | 33 | Adults |
| (Lutzenberger et al., 1982) | 1982 | Student volunteers | 20 | Adults |
| (Lutzenberger et al., 1993) | 1993 | Student volunteers | 30 | Range: 20-35 |
| (Mohr et al., 1998) | 1998 | Healthy subjects | 12 | Range: 23-65 |
| (Pauli et al., 1998) | 1998 | n/s | 11 | M = 24.9, SD = 2.3 |
| (Roberts et al., 1989) | 1989 | Student volunteers | 22 | Adults |
| (Rockstroh et al., 1990) | 1990 | Student volunteers | 45 and 48 (two experiments) | Adults |
| (Rockstroh et al., 1980) | 1980 | Student volunteers | 20 | Adults |
| (Rockstroh et al., 1982) | 1982 | Student volunteers | 20 | Adults |
| (Schneider et al., 1993) | 1993 | Alcohol dependency | 10 | M = 35.2 (range: 24-52) |
| (Schneider et al., 1992a) | 1992 | Depression, healthy subjects | 16 | Depression: M = 47.8 (range: 38-56)  Healthy: M = 38.5 (range: 30-55) |
| (Schneider et al., 1992b) | 1992 | Schizophrenia, healthy subjects | 24 | Schizophrenia: M = 27.3 (range: 20-32)  Healthy: M = 27.5 (range: 20-32) |
| (Daum et al., 1993) | 1993 | Epilepsy | 14 | M = 29.9 years (range 15-47) |
| (Rockstroh et al., 1993) | 1993 | Epilepsy | 25 | M = 30.1, SD = 1.8 (range: 15-49) |

**Note.** ALS: Amyotrophic lateral sclerosis; M: Mean, n/s: Not specified; SD: Standard deviation

| **Supplementary Table S3a.** Scores based on CRED-nf checklist and SCP-NF specific items (items 8 & 9). | | | | | | | | | | | | | | | | | | | | | | | | | | | | | |
| --- | --- | --- | --- | --- | --- | --- | --- | --- | --- | --- | --- | --- | --- | --- | --- | --- | --- | --- | --- | --- | --- | --- | --- | --- | --- | --- | --- | --- | --- |
| **Pre-experiment** | | | **Control groups** | | | | | **Control measures** | | | | | **Feedback specifications** | | | | | **Outcome measures (Brain)** | | | **Outcome measures (Behaviour)** | | **Data storage** | | **Technical details** | | | **Acquisition and application** | |
|  | CRED-nf Checklist (for details see Ros et al., 2020) | | | | | | | | | | | | | | | | | | | | | | |  | SCP-NF specific addendum* | | | | |
| **Article** | **1a** | **1b** | **2a** | **2b** | **2c** | **2d** | **2e** | **3a** | **3b** | **3c** | **3d** | **3e** | **4a** | **4b** | **4c** | **4d** | **4e** | **5a** | **5b** | **5c** | **6a** | **6b** | **7a** |  | **8a** | **8b** | **8c** | **9a** | **9b** |
| Konicar et al., 2021a | 1 | 0 | 1 | 0 | 0 | 0 | 1 | 1 | 1 | 1 | 1 | 0 | 1 | 1 | 1 | 1 | 1 | 1 | 1 | 0 | 1 | 1 | 1 |  | 1 | 1 | 0 | 0 | 1 |
| Konicar et al., 2021b | 0 | 1 | 0 | 0 | 0 | 0 | 0 | 0 | 1 | 0 | 1 | 0 | 1 | 1 | 1 | 0 | 1 | 0 | 0 | 0 | 0 | 0 | 0 |  | 1 | 1 | 0 | 1 | 0 |
| Hasslinger et al., 2020 | 1 | 0 | 0 | 0 | 0 | 0 | 0 | 1 | 1 | 1 | 1 | 0 | 1 | 1 | 1 | 1 | 1 | 1 | 1 | 0 | 0 | 0 | 0 |  | 1 | 1 | 1 | 1 | 0 |
| Hasslinger et al., 2021 | 1 | 1 | 1 | 0 | 0 | 1 | 1 | 0 | 1 | 0 | 1 | 0 | 1 | 1 | 1 | 1 | 1 | 1 | 1 | 0 | 0 | 1 | 0 |  | 1 | 1 | 1 | 1 | 0 |
| Heinrich et al., 2020 | 1 | 1 | 1 | 0 | 0 | 0 | 0 | 0 | 1 | 0 | 1 | 0 | 1 | 1 | 1 | 0 | 1 | 0 | 0 | 0 | 0 | 0 | 0 |  | 1 | 1 | 0 | 0 | 1 |
| Krepler et al., 2020 | 0 | 0 | 0 | 0 | 0 | 0 | 0 | 0 | 0 | 0 | 0 | 0 | 0 | 0 | 0 | 0 | 1 | 0 | 0 | 0 | 1 | 0 | 0 |  | 0 | 0 | 0 | 0 | 0 |
| Aggensteiner et al., 2019 | 1 | 0 | 1 | 0 | 0 | 0 | 1 | 0 | 0 | 0 | 1 | 0 | 1 | 1 | 1 | 1 | 1 | 1 | 1 | 0 | 0 | 1 | 0 |  | 1 | 1 | 0 | 1 | 1 |
| Baumeister et al., 2019 | 1 | 0 | 1 | 0 | 0 | 0 | 0 | 0 | 1 | 0 | 1 | 0 | 1 | 1 | 1 | 0 | 0 | 0 | 0 | 0 | 0 | 0 | 0 |  | 1 | 1 | 0 | 1 | 1 |
| Morales-Quezada et al., 2019 | 0 | 0 | 1 | 1 | 1 | 0 | 1 | 0 | 0 | 0 | 1 | 0 | 1 | 1 | 1 | 0 | 1 | 0 | 0 | 0 | 0 | 0 | 0 |  | 1 | 0 | 0 | 0 | 0 |
| Okumura et al., 2019 | 0 | 0 | 0 | 0 | 0 | 0 | 0 | 0 | 0 | 0 | 1 | 0 | 1 | 1 | 1 | 1 | 1 | 1 | 1 | 0 | 0 | 0 | 0 |  | 1 | 1 | 1 | 0 | 0 |
| Baumeister et al., 2018 | 1 | 0 | 1 | 0 | 0 | 0 | 0 | 0 | 1 | 0 | 1 | 0 | 1 | 1 | 1 | 1 | 0 | 1 | 1 | 0 | 0 | 0 | 0 |  | 1 | 1 | 0 | 1 | 1 |
| Minder et al., 2018 | 1 | 1 | 1 | 0 | 1 | 1 | 1 | 0 | 0 | 0 | 0 | 0 | 0 | 0 | 0 | 0 | 1 | 0 | 0 | 0 | 1 | 0 | 0 |  | 1 | 1 | 1 | 0 | 0 |
| Zuberer et al., 2018 | 1 | 0 | 1 | 0 | 0 | 0 | 1 | 0 | 0 | 0 | 1 | 0 | 1 | 1 | 1 | 1 | 1 | 1 | 1 | 0 | 0 | 0 | 0 |  | 1 | 1 | 1 | 0 | 0 |
| Albrecht et al., 2017 | 0 | 1 | 0 | 0 | 0 | 0 | 0 | 0 | 1 | 0 | 1 | 0 | 1 | 1 | 1 | 1 | 1 | 1 | 1 | 0 | 0 | 0 | 0 |  | 1 | 1 | 1 | 1 | 1 |
| Strehl et al., 2017 | 1 | 1 | 1 | 0 | 1 | 0 | 1 | 0 | 0 | 0 | 1 | 0 | 1 | 1 | 1 | 1 | 1 | 1 | 1 | 1 | 1 | 1 | 0 |  | 1 | 1 | 0 | 1 | 1 |
| Mayer et al., 2016 | 1 | 0 | 0 | 0 | 0 | 0 | 0 | 1 | 0 | 0 | 1 | 0 | 1 | 1 | 1 | 1 | 1 | 1 | 0 | 1 | 0 | 0 | 0 |  | 1 | 1 | 0 | 1 | 1 |
| Milner et al., 2016 | 0 | 0 | 0 | 0 | 0 | 0 | 0 | 0 | 0 | 0 | 1 | 0 | 1 | 1 | 1 | 1 | 1 | 1 | 1 | 0 | 0 | 0 | 0 |  | 0 | 1 | 1 | 1 | 0 |
| Konicar et al., 2015 | 0 | 1 | 0 | 0 | 0 | 0 | 0 | 0 | 1 | 0 | 1 | 0 | 1 | 1 | 1 | 1 | 1 | 1 | 0 | 0 | 0 | 1 | 0 |  | 1 | 1 | 0 | 1 | 0 |
| Marx et al., 2015 | 1 | 1 | 1 | 0 | 0 | 0 | 1 | 0 | 0 | 0 | 1 | 0 | 1 | 1 | 1 | 0 | 1 | 0 | 0 | 0 | 0 | 0 | 0 |  | 1 | 1 | 0 | 1 | 0 |
| Christiansen et al., 2014 | 1 | 0 | 1 | 0 | 0 | 0 | 1 | 0 | 1 | 0 | 1 | 0 | 1 | 1 | 1 | 0 | 1 | 0 | 0 | 0 | 0 | 0 | 0 |  | 1 | 1 | 1 | 1 | 1 |
| Gevensleben et al., 2014a | 0 | 0 | 0 | 0 | 0 | 0 | 0 | 0 | 0 | 0 | 1 | 0 | 1 | 1 | 1 | 1 | 1 | 1 | 1 | 0 | 1 | 1 | 0 |  | 1 | 1 | 0 | 1 | 0 |
| Gevensleben et al., 2014b  experiment 1 | 0 | 0 | 1 | 0 | 0 | 0 | 0 | 0 | 1 | 0 | 1 | 0 | 1 | 1 | 1 | 0 | 1 | 0 | 0 | 0 | 0 | 0 | 0 |  | 1 | 0 | 0 | 1 | 1 |
| Gevensleben et al., 2014b  experiment 2 | 0 | 0 | 1 | 1 | 0 | 1 | 0 | 0 | 1 | 0 | 1 | 0 | 1 | 1 | 1 | 1 | 1 | 1 | 1 | 1 | 0 | 0 | 0 |  | 1 | 0 | 0 | 0 | 1 |
| Strehl et al., 2014 | 0 | 0 | 1 | 0 | 0 | 0 | 1 | 0 | 1 | 0 | 1 | 0 | 1 | 1 | 1 | 1 | 1 | 1 | 0 | 0 | 0 | 0 | 0 |  | 1 | 1 | 0 | 0 | 1 |
| Studer et al., 2014 | 0 | 0 | 1 | 0 | 0 | 0 | 0 | 0 | 1 | 0 | 1 | 0 | 1 | 1 | 1 | 0 | 1 | 1 | 0 | 1 | 0 | 0 | 0 |  | 1 | 1 | 0 | 0 | 1 |
| Takahashi et al., 2014 | 0 | 0 | 0 | 0 | 0 | 0 | 0 | 0 | 1 | 0 | 0 | 0 | 1 | 1 | 1 | 1 | 1 | 1 | 1 | 0 | 0 | 0 | 0 |  | 1 | 1 | 1 | 0 | 0 |
| Fumuro et al., 2013 | 0 | 0 | 1 | 0 | 0 | 0 | 0 | 0 | 1 | 0 | 1 | 0 | 1 | 1 | 1 | 1 | 1 | 1 | 0 | 0 | 0 | 0 | 0 |  | 1 | 1 | 0 | 0 | 0 |
| Mayer et al., 2012 | 0 | 0 | 1 | 0 | 0 | 0 | 0 | 0 | 0 | 0 | 1 | 0 | 1 | 1 | 1 | 0 | 1 | 0 | 0 | 0 | 0 | 0 | 0 |  | 1 | 1 | 0 | 0 | 0 |
| Strehl et al., 2011 | 0 | 0 | 1 | 0 | 0 | 0 | 1 | 0 | 0 | 0 | 1 | 0 | 1 | 1 | 1 | 0 | 1 | 0 | 0 | 0 | 0 | 0 | 0 |  | 1 | 1 | 0 | 0 | 0 |
| Wangler et al., 2011 | 1 | 1 | 1 | 0 | 1 | 1 | 0 | 0 | 1 | 0 | 1 | 0 | 1 | 1 | 1 | 0 | 1 | 0 | 0 | 1 | 0 | 0 | 0 |  | 1 | 1 | 0 | 0 | 1 |
| Gevensleben et al., 2010 | 1 | 1 | 1 | 0 | 0 | 0 | 0 | 0 | 1 | 0 | 1 | 0 | 1 | 1 | 1 | 0 | 1 | 0 | 0 | 0 | 1 | 0 | 0 |  | 1 | 1 | 0 | 0 | 1 |
| Spronk et al., 2010 | 0 | 0 | 1 | 0 | 0 | 0 | 0 | 0 | 1 | 0 | 1 | 0 | 1 | 1 | 1 | 1 | 1 | 1 | 1 | 1 | 0 | 0 | 0 |  | 1 | 1 | 1 | 0 | 0 |
| Gevensleben et al., 2009b | 1 | 1 | 1 | 0 | 0 | 0 | 0 | 1 | 1 | 0 | 1 | 0 | 1 | 1 | 1 | 0 | 1 | 0 | 0 | 0 | 1 | 0 | 0 |  | 1 | 1 | 0 | 0 | 1 |
| Gevensleben et al., 2009a | 1 | 1 | 1 | 0 | 0 | 0 | 0 | 1 | 1 | 0 | 1 | 0 | 1 | 1 | 1 | 0 | 1 | 0 | 0 | 0 | 0 | 0 | 0 |  | 1 | 1 | 0 | 0 | 0 |
| Doehnert et al., 2008 | 0 | 0 | 1 | 0 | 0 | 0 | 1 | 0 | 1 | 0 | 1 | 0 | 1 | 1 | 1 | 1 | 1 | 1 | 0 | 1 | 0 | 1 | 0 |  | 1 | 1 | 0 | 1 | 1 |
| Drechsler et al., 2007 | 0 | 0 | 1 | 0 | 0 | 0 | 1 | 0 | 1 | 0 | 1 | 0 | 1 | 1 | 1 | 1 | 1 | 1 | 1 | 1 | 0 | 1 | 0 |  | 1 | 1 | 0 | 1 | 1 |
| Kleinnijenhuis et al., 2007 | 0 | 0 | 1 | 0 | 0 | 0 | 0 | 0 | 1 | 0 | 1 | 0 | 1 | 1 | 1 | 1 | 1 | 1 | 1 | 1 | 0 | 0 | 0 |  | 1 | 1 | 1 | 1 | 0 |
| Leins et al., 2007 | 0 | 0 | 1 | 0 | 0 | 0 | 0 | 1 | 1 | 0 | 1 | 0 | 1 | 1 | 1 | 1 | 1 | 1 | 1 | 0 | 0 | 0 | 0 |  | 1 | 1 | 0 | 1 | 1 |
| Strehl et al., 2006a | 0 | 0 | 0 | 0 | 0 | 0 | 0 | 0 | 1 | 0 | 1 | 0 | 1 | 1 | 1 | 1 | 1 | 1 | 1 | 0 | 1 | 1 | 0 |  | 1 | 1 | 0 | 1 | 1 |
| Strehl et al., 2006b | 0 | 0 | 0 | 0 | 0 | 0 | 0 | 0 | 1 | 0 | 1 | 0 | 1 | 1 | 1 | 1 | 1 | 1 | 0 | 0 | 0 | 0 | 0 |  | 1 | 0 | 0 | 0 | 0 |
| Strehl et al., 2005 | 0 | 0 | 0 | 0 | 0 | 0 | 0 | 1 | 1 | 0 | 1 | 0 | 1 | 1 | 1 | 1 | 1 | 1 | 0 | 0 | 0 | 1 | 0 |  | 1 | 1 | 0 | 0 | 0 |
| Heinrich et al., 2004 | 0 | 0 | 1 | 0 | 0 | 0 | 1 | 0 | 1 | 0 | 1 | 0 | 1 | 1 | 1 | 0 | 1 | 0 | 0 | 1 | 0 | 0 | 0 |  | 1 | 1 | 0 | 0 | 1 |
| Hinterberger et al., 2003 | 0 | 0 | 0 | 0 | 0 | 0 | 0 | 0 | 1 | 1 | 1 | 0 | 1 | 1 | 1 | 1 | 0 | 1 | 0 | 0 | 0 | 0 | 0 |  | 1 | 0 | 0 | 0 | 0 |
| Kotchoubey et al., 2002 | 0 | 0 | 0 | 0 | 0 | 0 | 0 | 0 | 1 | 0 | 1 | 0 | 1 | 1 | 1 | 0 | 0 | 0 | 0 | 0 | 0 | 0 | 0 |  | 1 | 0 | 0 | 0 | 0 |
| Kotchoubey et al., 2001 | 0 | 0 | 1 | 0 | 0 | 0 | 1 | 1 | 1 | 0 | 1 | 0 | 1 | 1 | 1 | 0 | 0 | 0 | 0 | 0 | 0 | 0 | 0 |  | 1 | 1 | 0 | 0 | 1 |
| Uhlmann et al., 2001 | 0 | 0 | 1 | 0 | 0 | 0 | 0 | 0 | 0 | 0 | 0 | 0 | 0 | 1 | 1 | 0 | 0 | 0 | 0 | 0 | 0 | 0 | 0 |  | 1 | 0 | 1 | 0 | 0 |
| Kotchoubey et al., 2000 | 0 | 0 | 0 | 0 | 0 | 0 | 0 | 0 | 0 | 0 | 1 | 0 | 1 | 1 | 1 | 1 | 1 | 1 | 0 | 1 | 0 | 0 | 0 |  | 1 | 0 | 0 | 1 | 0 |
| Pulvermuller et al., 2000 | 0 | 0 | 0 | 0 | 0 | 0 | 0 | 0 | 1 | 1 | 1 | 0 | 1 | 1 | 1 | 1 | 1 | 1 | 0 | 1 | 0 | 0 | 0 |  | 1 | 0 | 0 | 1 | 0 |
| Siniatchkin et al., 2000b | 0 | 0 | 0 | 0 | 0 | 0 | 0 | 0 | 1 | 1 | 1 | 0 | 1 | 1 | 1 | 1 | 1 | 1 | 1 | 0 | 0 | 0 | 0 |  | 1 | 1 | 1 | 1 | 1 |
| Siniatchkin et al., 2000a | 0 | 0 | 1 | 0 | 1 | 0 | 0 | 0 | 1 | 0 | 1 | 0 | 1 | 1 | 1 | 1 | 1 | 1 | 1 | 1 | 1 | 1 | 0 |  | 1 | 1 | 1 | 1 | 0 |
| **Note.** The appraisal of the articles was solely focused on Slow Cortical Potential Neurofeedback, other neurofeedback protocols were not considered when scoring.  *****See table S3c for details on items. | | | | | | | | | | | | | | | | | | | | | | | | | | | | | |

| **Supplementary Table S3b.** Percentages per item of CRED-nf checklist and SCP-NF specific addendum. | | | | | | | | | | |
| --- | --- | --- | --- | --- | --- | --- | --- | --- | --- | --- |
|  | **Item 1** | **Item 2** | **Item 3** | **Item 4** | **Item 5** | **Item 6** | **Total** | **Item 8** | **Item 9** | **TOTAL incl addendum** |
| **Articles** | CRED-nf Checklist (for details see Ros et al., 2020) | | | | | | | SCP-NF specific addendum* | | |
| Konicar et al., 2021a | 50% | 40% | 80% | 100% | 67% | 100% | 73% | 67% | 50% | 69% |
| Konicar et al., 2021b | 50% | 0% | 40% | 80% | 0% | 0% | 28% | 67% | 50% | 36% |
| Hasslinger et al., 2020 | 50% | 0% | 80% | 100% | 67% | 0% | 49% | 100% | 50% | 56% |
| Hasslinger et al., 2021 | 100% | 60% | 40% | 100% | 67% | 50% | 69% | 100% | 50% | 71% |
| Heinrich et al., 2020 | 100% | 20% | 40% | 80% | 0% | 0% | 40% | 67% | 50% | 45% |
| Krepler et al., 2020 | 0% | 0% | 0% | 20% | 0% | 50% | 12% | 0% | 0% | 9% |
| Aggensteiner et al., 2019 | 50% | 40% | 20% | 100% | 67% | 50% | 54% | 67% | 100% | 62% |
| Baumeister et al., 2019 | 50% | 20% | 40% | 60% | 0% | 0% | 28% | 67% | 100% | 42% |
| Morales-Quezada et al., 2019 | 0% | 80% | 20% | 80% | 0% | 0% | 30% | 33% | 0% | 27% |
| Okumura et al., 2019 | 0% | 0% | 20% | 100% | 67% | 0% | 31% | 100% | 0% | 36% |
| Baumeister et al., 2018 | 50% | 20% | 40% | 80% | 67% | 0% | 43% | 67% | 100% | 53% |
| Minder et al., 2018 | 100% | 80% | 0% | 20% | 0% | 50% | 42% | 100% | 0% | 44% |
| Zuberer et al., 2018 | 50% | 40% | 20% | 100% | 67% | 0% | 46% | 100% | 0% | 47% |
| Albrecht et al., 2017 | 50% | 0% | 40% | 100% | 67% | 0% | 43% | 100% | 100% | 57% |
| Strehl et al., 2017 | 100% | 60% | 20% | 100% | 100% | 100% | 80% | 67% | 100% | 81% |
| Mayer et al., 2016 | 50% | 0% | 40% | 100% | 67% | 0% | 43% | 67% | 100% | 53% |
| Milner et al., 2016 | 0% | 0% | 20% | 100% | 67% | 0% | 31% | 67% | 50% | 38% |
| Konicar et al., 2015 | 50% | 0% | 40% | 100% | 33% | 50% | 46% | 67% | 50% | 49% |
| Marx et al., 2015 | 100% | 40% | 20% | 80% | 0% | 0% | 40% | 67% | 50% | 45% |
| Christiansen et al., 2014 | 50% | 40% | 40% | 80% | 0% | 0% | 35% | 100% | 100% | 51% |
| Gevensleben et al., 2014a | 0% | 0% | 20% | 100% | 67% | 100% | 48% | 67% | 50% | 50% |
| Gevensleben et al., 2014b  experiment 1 | 0% | 20% | 40% | 80% | 0% | 0% | 23% | 33% | 100% | 34% |
| Gevensleben et al., 2014b  experiment 2 | 0% | 60% | 40% | 100% | 100% | 0% | 50% | 33% | 50% | 48% |
| Strehl et al., 2014 | 0% | 40% | 40% | 100% | 33% | 0% | 36% | 67% | 50% | 41% |
| Studer et al., 2014 | 0% | 20% | 40% | 80% | 67% | 0% | 34% | 67% | 50% | 40% |
| Takahashi et al., 2014 | 0% | 0% | 20% | 100% | 67% | 0% | 31% | 100% | 0% | 36% |
| Fumuro et al., 2013 | 0% | 20% | 40% | 100% | 33% | 0% | 32% | 67% | 0% | 33% |
| Mayer et al., 2012 | 0% | 20% | 20% | 80% | 0% | 0% | 20% | 67% | 0% | 23% |
| Strehl et al., 2011 | 0% | 40% | 20% | 80% | 0% | 0% | 23% | 67% | 0% | 26% |
| Wangler et al., 2011 | 100% | 60% | 40% | 80% | 33% | 0% | 52% | 67% | 50% | 54% |
| Gevensleben et al., 2010 | 100% | 20% | 40% | 80% | 0% | 50% | 48% | 67% | 50% | 51% |
| Spronk et al., 2010 | 0% | 20% | 40% | 100% | 100% | 0% | 43% | 100% | 0% | 45% |
| Gevensleben et al., 2009b | 100% | 20% | 60% | 80% | 0% | 50% | 52% | 67% | 50% | 53% |
| Gevensleben et al., 2009a | 100% | 20% | 60% | 80% | 0% | 0% | 43% | 67% | 0% | 41% |
| Doehnert et al., 2008 | 0% | 40% | 40% | 100% | 67% | 50% | 49% | 67% | 100% | 58% |
| Drechsler et al., 2007 | 0% | 40% | 40% | 100% | 100% | 50% | 55% | 67% | 100% | 62% |
| Kleinnijenhuis et al., 2007 | 0% | 20% | 40% | 100% | 100% | 0% | 43% | 100% | 50% | 51% |
| Leins et al., 2007 | 0% | 20% | 60% | 100% | 67% | 0% | 41% | 67% | 100% | 52% |
| Strehl et al., 2006a | 0% | 0% | 40% | 100% | 67% | 100% | 51% | 67% | 100% | 59% |
| Strehl et al., 2006b | 0% | 0% | 40% | 100% | 33% | 0% | 29% | 33% | 0% | 26% |
| Strehl et al., 2005 | 0% | 0% | 60% | 100% | 33% | 50% | 41% | 67% | 0% | 39% |
| Heinrich et al., 2004 | 0% | 40% | 40% | 80% | 33% | 0% | 32% | 67% | 50% | 39% |
| Hinterberger et al., 2003 | 0% | 0% | 60% | 80% | 33% | 0% | 29% | 33% | 0% | 26% |
| Kotchoubey et al., 2002 | 0% | 0% | 40% | 60% | 0% | 0% | 17% | 33% | 0% | 17% |
| Kotchoubey et al., 2001 | 0% | 40% | 60% | 60% | 0% | 0% | 27% | 67% | 50% | 35% |
| Uhlmann et al., 2001 | 0% | 20% | 0% | 40% | 0% | 0% | 10% | 67% | 0% | 16% |
| Kotchoubey et al., 2000 | 0% | 0% | 20% | 100% | 67% | 0% | 31% | 33% | 50% | 34% |
| Pulvermuller et al., 2000 | 0% | 0% | 60% | 100% | 67% | 0% | 38% | 33% | 50% | 39% |
| Siniatchkin et al., 2000b | 0% | 0% | 60% | 100% | 67% | 0% | 38% | 100% | 100% | 53% |
| Siniatchkin et al., 2000a | 0% | 40% | 40% | 100% | 100% | 100% | 63% | 100% | 50% | 66% |
| **Note.** The appraisal of the articles was solely focused on Slow Cortical Potential Neurofeedback, other neurofeedback protocols were not considered when scoring.  ***** See table S3c for details on items. | | | | | | | | | | |

| **Supplementary Table S3c.** Details concerning SCP-NF specific items | |
| --- | --- |
| **Item 8 –** | **Technical details** |
| 8a | Report the number of trials and trial length. |
| 8b | Report the ratio of activation and deactivation trials. Also, the ratio of transfer- to feedback trials per training block should be reported. |
| 8c | Report if a threshold had to be surpassed for a trial to be classed as successful. |
| **Item 9 -** | **Acquisition and application** |
| 9a | Report and describe whether a reward system, beyond contingent feedback, was utilized. |
| 9b | Report and describe the use of any transfer promoting exercises, e.g., transfer cards. The simple utilization of transfer cards or instructing participants to practice strategies during breaks, does not suffice. Instead, transfer promoting activities should be implemented together with the trainer. |
|  | |
